# Pneumococcal responses are similar in Papua New Guinean children aged 3-5 years vaccinated in infancy with pneumococcal polysaccharide vaccine with or without prior pneumococcal conjugate vaccine, or without pneumococcal vaccination

**DOI:** 10.1371/journal.pone.0185877

**Published:** 2017-10-13

**Authors:** Anita H. J. van den Biggelaar, Peter C. Richmond, Angela Fuery, Denise Anderson, Christine Opa, Gerard Saleu, Mildred Lai, Jacinta P. Francis, Michael P. Alpers, William S. Pomat, Deborah Lehmann

**Affiliations:** 1 Telethon Kids Institute, University of Western Australia, Perth, Western Australia, Australia; 2 School of Paediatrics and Child Health, University of Western Australia, Perth, Western Australia, Australia; 3 Papua New Guinea Institute of Medical Research, Goroka, Eastern Highlands Province, Papua New Guinea; 4 International Health, School of Health Sciences, Curtin University, Perth, Western Australia, Australia; Public Health England, UNITED KINGDOM

## Abstract

**Trial design:**

In an earlier trial, Papua New Guinean (PNG) children at high risk of pneumococcal disease were randomized to receive 0 or 3 doses of 7-valent pneumococcal conjugate vaccine (PCV7), followed by a single dose of 23-valent pneumococcal polysaccharide vaccine (PPV23) at 9 months of age. We here studied in a non-randomized follow-up trial the persistence of pneumococcal immunity in these children at 3–5 years of age (n = 132), and in 121 community controls of a similar age with no prior pneumococcal vaccination.

**Methods:**

Circulating IgG antibody titers to all PCV7 and PPV23-only serotypes 2, 5 and 7F were measured before and after challenge with 1/5^th^ of a normal PPV23 dose. Serotype-specific memory B-cells were enumerated at 10 months and 3–5 years of age for a subgroup of study children.

**Results:**

Serotype-specific IgG antibody titers before and after challenge were similar for children who received PCV7/PPV23, PPV23 only, or no pneumococcal vaccines. Before challenge, at least 89% and 59% of children in all groups had serotype-specific titers **≥** 0.35μg/ml and **≥** 1.0 μg/ml, respectively. Post-challenge antibody titers were higher or similar to pre-challenge titers for most children independent of pneumococcal vaccination history. The rise in antibody titers was significantly lower when pre-challenge titers were higher. Overall the relative number of serotype-specific memory B-cells remained the same or increased between 10 months and 3–5 years of age, and there were no differences in serotype-specific memory B-cell numbers at 3–5 years of age between the three groups.

**Conclusions:**

Immunity induced by PCV7 and/or PPV23 immunization in infancy does not exceed that of naturally acquired immunity in 3-5-year-old children living in a highly endemic area. Also, there was no evidence that PPV23 immunization in the first year of life following PCV7 priming induces longer-term hypo-responsiveness.

**Trial registration:**

Clinicaltrials.gov NCT01414504 and NCT00219401.

## Introduction

*Streptococcus pneumoniae* remains a leading cause of death in children under 5 years of age, causing over 500,000 deaths and nearly 14 million episodes of disease annually [1]. Most cases occur in low-income countries in children younger than 6 months of age [1, 2]. In high-risk settings, infants are subject to pneumococcal colonization and disease at a younger age than children in low-risk settings [2], and the spectrum of pneumococcal serotypes carried and causing invasive disease is much broader [3, 4]. Preventing pneumococcal disease in children in high-risk settings therefore requires strategies that induce the earliest possible protection against the broadest spectrum of invasive pneumococcal serotypes possible.

More than 90 pneumococcal serotypes have been defined on the basis of the composition of the pneumococcal polysaccharide capsule. The capsular polysaccharide is the main virulence factor of the bacterium and immune responses to the capsule are serotype-specific. A licensed pneumococcal polysaccharide vaccine including 23 different pneumococcal serotypes (PPV23) is known to be effective in adults; however, PPV23 does not induce immunologic memory and its immunogenicity in young infants in general is believed to be limited [5] although PPV was shown to be efficacious against death due to pneumonia in young children in a high-risk setting, namely the Papua New Guinea (PNG) highlands. A 7-valent pneumococcal conjugate vaccine (PCV7) was licensed in 2000. PCVs are highly immunogenic in infants, induce immunological memory and serum antibodies with a strong opsonophagocytic (bacterial killing) capacity [6, 7]. Although the spectrum of serotypes against which PCVs can protect has improved with the licensure of higher valency vaccines (PCV10 and PCV13) in 2010, overall coverage is still limited in high-risk settings. For example, in a longitudinal cohort of newborns in a highly endemic setting in PNG we identified no less than 63 different pneumococcal serotypes in 1761 nasopharyngeal swabs, and only 43% of the pneumococcal isolates were of serotypes present in PCV13 [3].

This neonatal cohort study was part of a randomized controlled vaccination trial to determine the safety and immunogenicity of a neonatal (0-1-2 months) versus infant (1-2-3 months) immunization schedule of PCV7 [8]. The trial included a control arm not receiving PCV7. All children in the trial received a single dose of PPV23 at 9 months of age. In an earlier trial, a 45% protective effect of PPV against moderate/severe acute lower respiratory infections (ALRI) was found in young children in PNG at the time of an epidemic [9]; a 50–59% efficacy against ALRI mortality [10]; and an age-related induction of antibody responses against some serotypes frequently causing invasive disease consistent with efficacy trial results [11]. We have reported that PCV7 was safe in our neonatal cohort and that pneumococcal serotype-specific IgG antibody titers and T-cell responses measured shortly after completion of the 3 priming doses were similar for the neonatal and infant schedules [12, 13]. In PCV7-vaccinated infants, PPV23 induced significantly higher PCV7 serotype-specific antibody responses than in PCV7-unvaccinated infants, with no differences between neonatal and infant groups [13]. At completion of the trial when infants were 18 months of age, antibody concentrations had returned to pre-PPV23 levels in the PCV7-vaccinated groups and were comparable to those vaccinated with PPV23 only [13].

We now aimed to study the longer term immune protection induced by vaccination with PCV7 followed by PPV23 in these Papua New Guinean children. Since the circulation of pneumococci, including PCV7 and non-PCV7 serotypes, in this endemic setting is high [3], children are expected to develop naturally acquired immunity to a broad spectrum of serotypes. We therefore recruited a group of community controls of a similar age who had not received any pneumococcal vaccination and lived in the same area as the vaccinated study children in order to assess and compare levels of pneumococcal immunity. Since long-term individual protection against *Streptococcus pneumoniae* may depend on persisting antibody as well as B-cell memory recall responses, we measured serotype-specific antibody responses before and after a challenge with a low dose of PPV23, and assessed the number of serotype-specific memory B-cells when children were 10 months and 3–5 years old. Based on the capacity of PCVs to induce memory immune responses, we hypothesized that responses would be higher in children previously vaccinated with PCV7.

## Methods

### Study participants and previous vaccination trial

Recruitment for this study (NCT01414504) was conducted between May 25, 2010 and June 9, 2011, with follow-up of study participants taking place between June 23, 2010 and July 8, 2011. Study participants were children aged 3 to 5 years of age who had previously participated in an open randomized controlled neonatal PCV7 vaccination trial (2004–2009) (NCT00219401) conducted in the Asaro Valley in the Eastern Highlands Province of PNG, and community controls in the same age range who had not received any pneumococcal vaccination (pneumococcal vaccines were not routinely available at the time of the neonatal PCV7 trial and this follow-up study).

Inclusion criteria for children previously participating in the neonatal PCV7 study were: receipt of 0 or 3 doses of PCV7 according to protocol at 0, 1 and 2 months (neonatal schedule), or at 1, 2 and 3 months of age (infant schedule), and one dose of PPV23 between 9 and 12 months of age; and re-consent obtained from parents/guardians. Eligibility criteria for community controls were: age between 3 and 5 years at the time of interview; children from same communities as PCV7 trial participants; had not received any pneumococcal vaccines; and informed consent obtained. Children were excluded if they were known to be HIV positive or had other immunosuppressive conditions or treatments. Where possible, local village reporters re-established contact with families of eligible children who had previously participated in the PCV7 study, identified new places of residence if families had relocated, and provided initial information and pamphlets about the study. With the help of study families and other community members, local reports identified children aged 3–5 years in the community who would be eligible for recruitment as community controls. Next, study nurses visited eligible families to provide further information about the study, obtain assent and arrange a date for potential participants to visit the study clinic to complete informed consent and enrolment.

A detailed description of the earlier neonatal PCV7 vaccination trial can be found elsewhere (http://clinicaltrials.gov/ct2/show/NCT00219401) [8]. Briefly, a total of 318 newborns were enrolled and randomised to receive PCV7 (Prevnar7®, Wyeth, including pneumococcal serotypes 4, 6B, 9V, 14, 18C, 19F, 23F) within 3 days of birth, 1 and 2 months (neonatal group, n = 104), at 1, 2 and 3 months (infant group, n = 105), or no PCV7 (control group, n = 109). At 9 months of age all children received a single dose of PPV23 (Pneumovax®23, Merck & Co, containing capsular polysaccharides of serotypes 1, 2, 3, 4, 5, 6B, 7F, 8, 9N, 9V, 10A, 11A, 12F, 14, 15B, 17F, 18C, 19F, 19A, 20, 22F, 23F and 33). In addition, all children received routine childhood immunizations according to the PNG national immunization program. At 4 and 10 months of age venous blood samples were collected from all children to measure serum pneumococcal serotype-specific IgG antibody levels and isolate and cryopreserve peripheral blood mononuclear cells (PBMCs) for later cellular immunological experiments. Children were followed up until they were 18 months old.

The follow-up study presented here is registered under NCT01414504 (http://clinicaltrials.gov). The Trend Statement Checklist can be found in S1 Text.

### Ethical considerations

Ethical approvals for the follow-up study were obtained from the Institutional Review Board of the PNG Institute of Medical Research (reference number 0927) and PNG Medical Research Advisory Committee (MRAC, reference number 10.10). Written informed consent for participation in this follow-up study was obtained from all parents or guardians. This trial was conducted in compliance with the ICH principles of Good Clinical Practice (GCP) and the PNGIMR and NHMRC guidelines for clinical trials.

### Objectives

The aim of this study is to determine whether PPV23 given at 9 months of age (Protocol in S2 Text):

1. provides enhanced persistence of antibody levels associated with protection from invasive disease at 3 to 5 years of age compared to unvaccinated controls;
2. has an impact on the development of serotype -specific B-cell memory at age 3 to 5 years; and
3. enhances antibody persistence and B-cell memory for those serotypes included in PCV7 among children who received PCV7 in early infancy.

### Outcomes

The primary outcomes of this study were to determine the serotype-specific serum IgG antibody geometric mean concentrations (GMC) pre- and 1month post-challenge with a 0.1mL dose of PPV23 at 3–5 years of age for serotypes contained in PCV7 (4, 6B, 9V, 14, 18C, 19F, 23F) and PPV23 serotypes 1, 2, 3, 5, 7F, 19A; and the proportion of children with serotype-specific serum IgG antibody levels ≥ 0.35 μg/ml and ≥ 1.0 μg/ml before and after a PPV23 low-dose challenge for the above serotypes.

### Study procedures

During the first study visit, venous blood (up to 9 ml) was collected from all children prior to receiving a 0.1mL dose (1/5^th^ the normal dose) of PPV23 (Pneumovax®23, Merck & Co) as a single intramuscular dose into the deltoid muscle, equating to 5 μg of polysaccharide of each of the following serotypes: 1, 2, 3, 4, 5, 6B, 7F, 8, 9N, 9V, 10A,11A,12F, 14, 15B, 17F, 18C, 19A, 19F, 20, 22F, 23F, 33F. A second blood sample (up to 9 ml) was collected 1 month (21–56 days) post-challenge. All procedures were conducted by qualified research nurses during scheduled visits of study participants at the research clinic of the Papua New Guinea Institute of Medical Research (IMR) in Goroka, Papua New Guinea.

### Serum collection and isolation of peripheral mononuclear cells (PBMC)

Venous blood samples were collected into sterile 2 ml empty tubes (serum) and 10 ml tubes containing 100 IU preservative-free heparin (PBMC). Samples were centrifuged within 2 hours after collection to separate serum/plasma and aliquots were stored at -20°C. Where sufficient blood volume was available, PBMCs were isolated from the remaining heparin tube cell pellet by centrifugation over a Ficoll-Hypaque gradient (Lymphoprep, Axis-Shield, Oslo, Norway) and cryo-preserved in 50% heat-inactivated (HI) foetal calf serum (FCS) and 7.5% DMSO. Cells were kept under liquid nitrogen vapour phase conditions during storage at IMR, transport to and storage at the Telethon Kids Institute (formerly Telethon Institute of Child Health Research, ICHR) in Perth, Western Australia.

### Measurements of antibody concentrations

Serotype-specific antibody to PCV7/PPV23 serotypes 4, 6B, 9V, 14, 18C, 19F and 23F and PPV23 serotypes 2, 5 and 7F were measured using a standardized WHO ELISA with 22F adsorption in serum samples collected before and after challenge [13]. Data are available in S1 Data. Geometric mean antibody concentrations were calculated, as well as proportions of children with antibody titers above ≥0.35 μg/ml and ≥1.0 μg/ml: 0.35 μg/ml is considered the serologic correlate of protection against invasive pneumococcal disease following PCV immunization [14], while ≥1.0 μg/ml is a more conservative cut-off.

### Measurement of memory B-cells

Serotype-specific memory B-cells were measured by ELISPOT as described elsewhere [15, 16]. Briefly, PBMCs (2 x 10^6^) were cultured with polyclonal stimuli for 6 days to enable differentiation of memory B-cells into antibody forming cells (AFC). After culture, cells were seeded onto plates pre-coated with polysaccharides (10 μg/mL) for serotypes 1, 2, 5, 6B, 7F, 14, 19A, 19F and 23F which commonly cause invasive pneumococcal disease [4]. Antigen-specific memory B-cells were detected by spot formation that indicated AFC and these were expressed as number of AFC/1x10^6^ PBMCs.

### Sample size

The sample size for follow-up of children who previously participated in the neonatal PCV7 trial and received PPV23 at 9 months of age was limited by the number of children available for follow-up: it was estimated that approximately 75 children for each study arm in the earlier trial (i.e. Neonatal PCV7; Infant PCV; Controls) could be re-enrolled. It was calculated that this number provided the study 80% power to detect a 64% difference (2-sided test at p<0.05) in pneumococcal serotype-specific IgG antibody GMCs between either of the vaccinated groups (N = 75) and the control group (N = 150). This between-group-difference is applicable to either pre- or post- challenge data. A standard deviation in log GMC concentration of 1.25 (based on 18-month antibody data from the neonatal PCV study) was used in this calculation. The proposed sample sizes provided for a range of between group detectable differences (2-sided test at p<0.05) in seroprotection rate (IgG ≥ 1 μg/ml). For a serotype with a 10% seroprotection rate in the controls either pre- or post-challenge, the study was powered to detect an increase to 26% seroprotection in either vaccinated group. For a 50% seroprotection rate in controls the study was powered to detect an increase to 70% seroprotection in either vaccinated group.

### Statistical analysis

Geometric mean concentrations (GMCs) and 95% confidence intervals were calculated for serotype-specific IgG titers (S1 Data). Using IBM SPSS Statistics 23.0, differences in GMCs between groups were analyzed using Mann-Whitney U t-test, and Wilcoxon signed ranked test was used to test for differences in GMCs between time points within groups. Differences in proportions between groups were tested using Chi-square test or Fisher’s Exact-test. Using R 3.0.1 [17], the R package ‘gee’ [18] was used for implementation of generalised estimating equations (GEE) to estimate generalized linear model coefficient estimates of the association between (a) logged pre-challenge antibody titers and pneumococcal vaccine history; and (b) the logged difference of post-challenge minus pre-challenge responses and pneumococcal vaccine history and pre-challenge antibody titers. Models were adjusted for age, gender and clustering of children within the same household (8 pairs of children). Differences and associations were considered statistically significant if p < 0.05.

## Results

### Population characteristics

**Fig 1:**
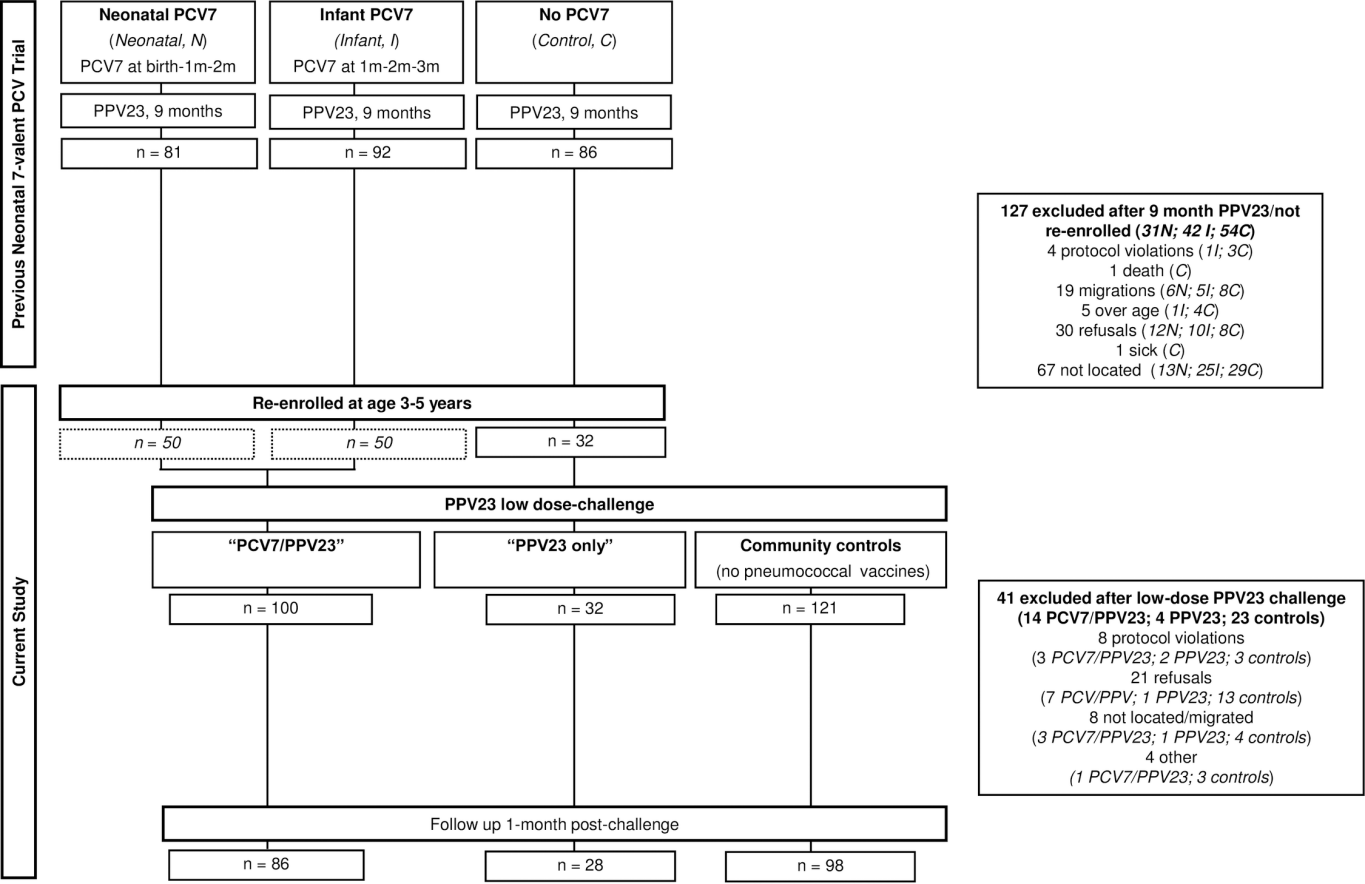
Flowchart for children in the study. A flowchart of the completed preceding neonatal PCV7 trial is published elsewhere [8]. A total of 132 of 259 children who participated in the previous PCV7 study and who were vaccinated with PPV23 at 9 months of age were included in this study. One hundred and twenty seven children could not be enrolled for reasons summarized in Fig 1, including death; being too old according to pre-defined inclusion criteria; illness; migration to an area outside the study’s reach capacities; not located; and refusal. A total of 121 of 136 potential community controls who were assented to participate in the study were included in the final analysis: of the 15 not included, three children were over age; three did not consent; three could not be relocated and blood collection pre-challenge was not successful for six children. Post-challenge data were not available for 41 children for reasons summarized. *N* is Neonatal PCV7 group; *I* is Infant PCV7 group; *C* is control group (no PCV7).

Follow-up of children who had participated in the original neonatal PCV7 trial resulted in the inclusion of 100 children into the ‘PCV7 and PPV23’ group’ who as part of the neonatal trial had received 3 doses of PCV7 (50 vaccinated with PCV7 according to a neonatal 0-1-2- month schedule and 50 according to an infant 1-2-3 month schedule) and a single dose of PPV23 at 9 months of age (‘PCV7 and PPV23’ group), and 32 children into the ‘PPV23 only’ group who as part of the neonatal trial had received a single dose of PPV23 at 9 months of age but no prior PCV7 (Fig 1). In addition, 121 controls who had never received pneumococcal vaccine were enrolled from the same communities as the children who had participated in the earlier trial. There were thus three groups in the final analysis: PCV7 and PPV23; PPV23 only; and no pneumococcal vaccine.

Children in the three groups were of comparable age at the time of enrolment, with a mean age of 4 years and 1 month (standard deviation (sd) 1.3 months) in the group with no pneumococcal vaccines, 4 years (sd 6.7 months) in the PCV7 and PPV23 group, and 4 years and 1 month (sd 8.1 months) in the PPV23-only group. While the gender distribution was comparable between community controls (no pneumococcal vaccines) (67/121, 55% boys) and children who participated previously in the neonatal PCV7 trial (74/132, 56%) (Chi-square test, p = 0.912), there were significantly more boys in the PPV23-only group (23/32, 72%) than in the group vaccinated with PCV7 and PPV23 (51/100, 51%) (Chi-square test, p = 0.038). This is consistent with the overall enrolment into the neonatal PCV7 trial [13].

### Serum pneumococcal antibody responses at 3–5 years of age prior to challenge

**Fig 2:**
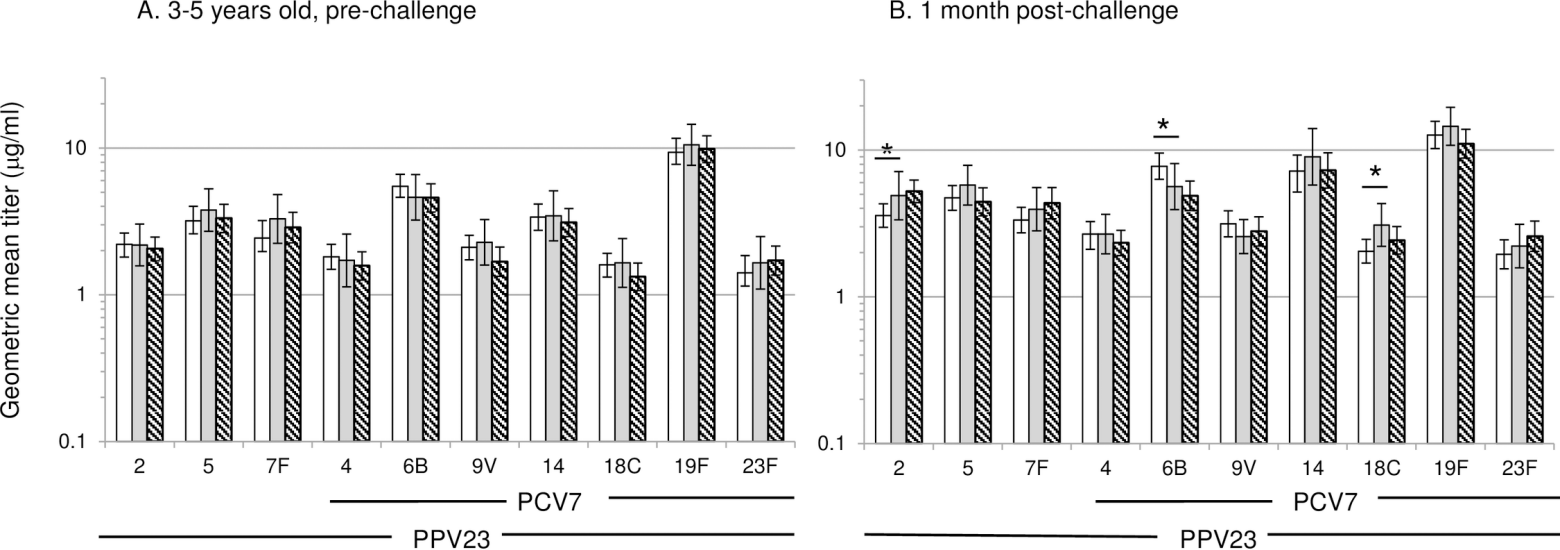
Pneumococcal serotype-specific IgG antibody titers before and after challenge. Serum antibody titers were assessed in 3-5-year-old children who had received PCV7 and PPV23 as infants (white bars; n = 100 /n = 86), who had received only PPV23 as infants (grey bars; n = 32 /n = 28), or who had not received pneumococcal vaccines (striped bars; n = 121 pre-challenge/ n = 98 post-challenge) (A) before and (B) after challenge with a low dose of PPV23. Data are presented as geometric mean titers and 95% confidence intervals. Serotype-specific geometric mean titers before or after challenge were compared between each group using Mann-Whitney U test. * p < 0.05.

At 3–5 years of age, circulating IgG antibody titers against all tested serotypes were similar for children in the three groups (Fig 2A). Generalised estimating equations (GEE) analysis that accounted for clustering of 8 pairs of children within the same household and adjusted for potential confounding by age and gender confirmed that there was no significant association of prior PPV23 (S1 Table) and PCV7 vaccination (S2 Table) with pre-challenge serotype-specific antibody titers. Increasing age was found to be significantly associated with increasing pre-challenge serotype-specific antibody concentrations for most serotypes.

For each measured serotype at least 88% of children in each treatment group had titers of **≥**0.35 μg/ml and at least 59% had titers of **≥**1.0 μg/ml (Table 1). In all three treatment groups more than 75% of the children had titers of **≥**1.0 μg/ml for six out of the ten tested serotypes. There were no significant differences in the proportions of children with serotype-specific IgG antibody titers of ≥0.35 μg/ml or ≥1.0 μg/ml, with the exception of serotype 4 for which the proportion of children with titers ≥1 μg/ml was significantly higher in children previously vaccinated with PPV23-only than in those who had not received any pneumococcal vaccines (p = 0.043) (Table 1).

**Table 1:** Proportion of 3-5-year-old children with pneumococcal serotype IgG antibody titers ≥ 0.35 μg/ml and ≥ 1 μg/ml before challenge.

	≥ 0.35 μg/ml			≥ 1 μg/ml			
	No vaccine (n = 121)	PCV7/PPV23 (n = 100)	PPV23 (n = 32)	No vaccine (n = 121)	PCV7/PPV23 (n = 100)	PPV23 (n = 32)	
2*	114 (94%)	97 (97%)	30 (94%)	92 (76%)	73 (73%)	27 (84%)	
5*	119 (98%)	98 (98%)	31 (97%)	102 (84%)	87 (87%)	30 (94%)	
7F*	114 (94%)	97 (97%)	30 (94%)	97 (80%)	80 (80%)	30 (94%)	
**4**	109 (90%)	95 (95%)	30 (94%)	71 (59%)	69 (69%)	25 (78%)	#
**6B**	119 (98%)	100 (100%)	32 (100%)	111 (92%)	96 (96%)	30 (94%)	
**9V**	109 (90%)	97 (97%)	30 (94%)	84 (69%)	77 (77%)	26 (81%)	
**14**	117 (97%)	99 (99%)	31 (97%)	103 (85%)	87 (87%)	25 (78%)	
**18C**	106 (88%)	94 (94%)	30 (94%)	77 (64%)	63 (63%)	25 (78%)	
**19F**	119 (98%)	100 (100%)	32 (100%)	118 (98%)	98 (98%)	32 (100%)	
**23F**	110 (91%)	92 (92%)	30 (94%)	77 (64%)	59 (59%)	21 (66%)	

*) Serotypes included in PPV23 but not PCV7.

Differences in proportions of children with serotype-specific IgG titers ≥ 0.35 μg/ml and ≥ 1 μg/ml between groups were tested using Chi-square test or Fisher’s Exact-test.

#) For serotype 4, more children previously vaccinated with PPV23-only had titers ≥ 1 μg/ml than children with no prior pneumococcal vaccination (p = 0.043).

### Post-challenge serum pneumococcal antibody responses

Children were challenged with a low dose of PPV23 and serum IgG antibodies were measured one month later. Post-challenge (geometric mean) antibody titers were generally comparable between the three groups (Fig 2B), with the exception of serotypes 2, 6B and 18C: children previously vaccinated with PCV7 and PPV23 showed lower titers to serotype 2 (p = 0.010) and higher titers to serotype 6B (p = 0.006) than children with no prior pneumococcal vaccination; and titers to serotype 18C were higher in children vaccinated with PPV23 only than in children vaccinated with both PCV7 and PPV23 (p = 0.030). As found before challenge, the proportions of children with serotype-specific IgG antibody titers ≥ 0.35 μg/ml or ≥ 1 μg/ml were similar in the three treatment groups after challenge, with the exception of serotype 2 for which the proportion of children with titers ≥1μg/ml was significantly higher in the previously unvaccinated group than in the group vaccinated with PCV7 and PPV23 (p = 0.047) and in the group vaccinated with PPV23 alone (p = 0.072) (Table 2).

**Table 2:** Proportion of 3-5-year-old children with pneumococcal serotype IgG antibody titers ≥ 0.35 μg/ml and ≥ 1 μg/ml one month after low-dose PPV23 challenge.

	≥ 0.35 μg/ml			≥ 1 μg/ml			
	No vaccine (n = 98)	PCV7/PPV23(n = 86)	PPV23(n = 28)	No vaccine (n = 98)	PCV7/PPV23(n = 86)	PPV23(n = 28)	
2*	98 (100%)	86 (100%)	28 (100%)	96 (98%)	78 (91%)	25 (89%)	#
5*	98 (100%)	86 (100%)	28 (100%)	89 (91%)	82 (95%)	28 (100%)	
7F*	97 (99%)	85 (99%)	28 (100%)	87 (89%)	80 (93%)	25 (89%)	
**4**	96 (98%)	85 (99%)	27 (96%)	83 (85%)	77 (90%)	24 (86%)	
**6B**	98 (100%)	86 (100%)	28 (100%)	91 (93%)	85 (99%)	28 (100%)	
**9V**	96 (98%)	85 (99%)	28 (100%)	83 (85%)	79 (92%)	26 (93%)	
**14**	97 (99%)	86 (100%)	28 (100%)	92 (94%)	84 (98%)	28 (100%)	
**18C**	93 (95%)	84 (98%)	28 (100%)	79 (81%)	69 (80%)	25 (89%)	
**19F**	98 (100%)	86 (100%)	28 (100%)	95 (97%)	85 (99%)	28 (100%)	
**23F**	96 (98%)	83 (97%)	27 (96%)	76 (78%)	62 (72%)	24 (86%)	

*) Serotypes included in PPV23 but not in PCV7.

Differences in proportions of children with serotype-specific IgG titers ≥ 0.35 μg/ml and ≥ 1 μg/ml between groups were tested using Chi-square test or Fisher’s Exact-test.

#) For serotype 2, more children not previously vaccinated with pneumococcal vaccines had titers ≥ 1 μg/ml than children previously vaccinated with PCV7 and PPV23 (p = 0.047).

**Fig 3:**
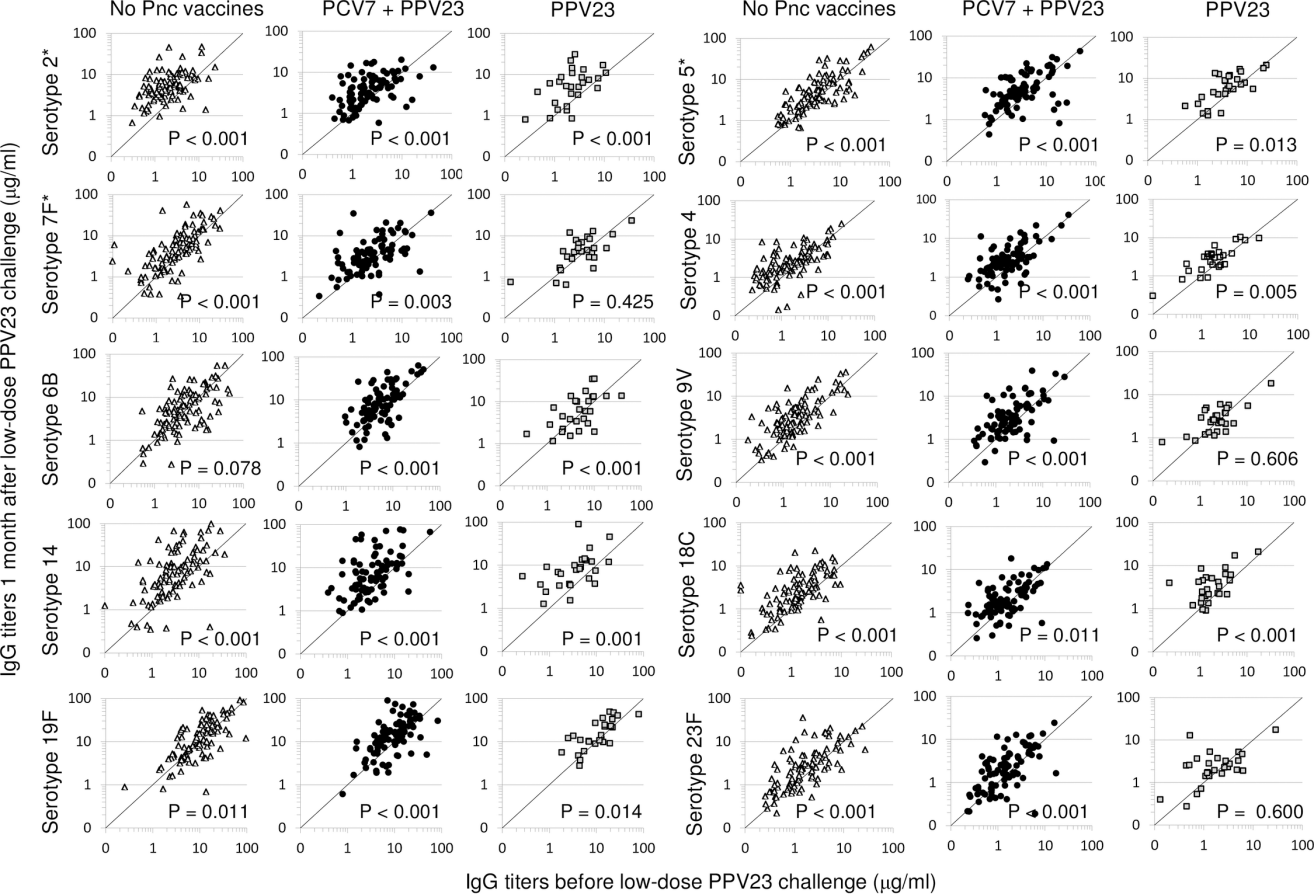
Individual pneumococcal serotype-specific IgG antibody titers before and after challenge. Graphs present scatterplots of pneumococcal serotype-specific IgG antibody titers pre- and 1 month post-challenge with a low dose of PPV23 for individual children in the groups not vaccinated with any pneumococcal vaccines (grey triangles), vaccinated with PCV7 and PPV23 (black circles), and vaccinated with PPV23 only (open squares). Serotypes with an asterisk (*) are included in PPV23 only, while other serotypes are included in both PCV7 and PPV23. For children whose antibody titers are higher post- than pre-challenge (increase in antibody titer) data points lie above the line; for children with similar titers before and after challenge data points cluster around the reference line; and for children whose antibody titers were lower 1month after challenge than before data points lie under the reference line. The p-values included in the graphs correspond with non-parametric analysis of paired pre- and post-challenge responses within the treatment groups (Wilcoxon Signed Rank Test).

When individual pre- and post-challenge titers were compared, the majority of children previously vaccinated with PCV7 and PPV23 showed significantly higher post-challenge antibody titers than pre-challenge titers to all tested vaccine serotypes (Fig 3). The same was found for unvaccinated children, except for response to serotype 6B for which the increase was not statistically significant. In children vaccinated with PPV23 only, antibody titers to serotypes 7F, 9V and 23F did not show a significant change, but increased significantly for the other seven tested serotypes.

Using generalised estimating equations (GEE) (Table 3), increased pre-challenge serotype-specific antibody concentrations were found to be associated with a decreased response to challenge. The geometric mean response to challenge was between 2% (95% CI: 1–3%; Serotype 19F) and 5% (95% CI: 4–6%; Serotype 2) lower for a 10% increase in pre-challenge serotype-specific antibody concentrations. An increase in age was found to be associated with an increased response to challenge for serotypes 14, 18C and 23F: for a 1 year increase in age, geometric mean responses to challenge were found to increase by 32% (95% CI: 12–57%) for serotype 14, 17% (95% CI: 1–35%) for serotype 18C, and 18% (95% CI: 1–36%) for serotype 23F. Boys showed a 19% (95% CI: 2–23%) lower geometric mean response to challenge for serotype 19F than girls, and a 23% (95% CI: 5–38%) lower response to challenge for serotype 23F.

**Table 3:** Generalised estimating equations studying associations between change in serotype-specific antibody titers in response to challenge and pneumococcal vaccine history and pre-challenge antibody titers.

Serotype	S 2	S 4	S 5	S 6B	S 7F	S 9V	S 14	S 18C	S 19 F	S 23 F
	$\hat{\beta }$(95% CI)	$\hat{\beta }$(95% CI)	$\hat{\beta }$(95% CI)	$\hat{\beta }$(95% CI)	$\hat{\beta }$(95% CI)	$\hat{\beta }$(95% CI)	$\hat{\beta }$(95% CI)	$\hat{\beta }$(95% CI)	$\hat{\beta }$(95% CI)	$\hat{\beta }$(95% CI)
**Pre-challenge IgG**	0.95(0.94–0.96)	0.96(0.95–0.97)	0.97(0.95–0.98)	0.97(0.96–0.98)	0.96(0.95–0.97)	0.97(0.96–0.98)	0.97(0.95–0.98)	0.96(0.95–0.97)	0.98(0.97–0.99)	0.97(0.96–0.98)
**Vaccination**										
None	1	1	1	1	1	1	1	1	1	1
PPV23	0.90(0.64–1.28)	1.13(0.91–1.41)	1.20(0.93–1.55)	1.18(0.85–1.64)	0.88(0.65–1.18)	0.75(0.58–0.97)	1.22(0.82–1.80)	1.18(0.88–1.57)	1.28(1.01–1.63)	0.93(0.67–1.31)
PCV7/PPV23	0.66(0.53–0.82)	1.06(0.86–1.29)	1.09(0.89–1.34)	1.42(1.13–1.80)	0.85(0.67–1.08)	0.97 (0.77–1.21)	0.97(0.72–1.30)	0.76(0.62–0.94)	1.18(0.96–1.46)	0.85(0.67–1.08)
**Gender**										
Female	1	1	1	1	1	1	1	1	1	1
Male	0.98	0.86	0.89	1.00	0.91	0.94	1.07	0.83	0.81	0.77
	(0.81–1.20)	(0.72–1.03)	(0.74–1.08)	(0.81–1.24)	(0.73–1.14)	(0.77–1.14)	(0.81–1.42)	(0.68–1.01)	(0.67–0.98)	(0.62–0.95)
**Age** (years)	0.95(0.84–1.09)	1.06(0.93–1.20)	1.14(1.00–1.29)	1.08(0.94–1.25)	1.15(0.99–1.33)	1.04(0.91–1.19)	1.32(1.12–1.57)	1.17(1.01–1.35)	1.14(1.00–1.29)	1.18(1.01–1.36)
**Interval**(days)	1.00(0.99–1.01)	1.00(0.98–1.01)	1.00(1.00–1.01)	0.99(0.98–1.01)	0.99(0.98–1.01)	1.00(0.99–1.01)	0.99(0.97–1.00)	1.00(0.99–1.00)	1.00(0.99–1.01)	0.99(0.98–1.01)

Generalised estimating equations (GEE) were applied to study correlations between (log-transformed) differences in serotype-specific antibody concentrations after and before challenge (IgG post-challenge−IgG _pre-challenge_) and (log-transformed) pre-challenge serotype-specific antibody concentrations (IgG _pre-challenge_), pneumococcal vaccination status, gender, age prior to challenge, and number of days between venous blood collection for assessment of pre- and post-challenge antibody titers (‘interval’). Generalized linear model coefficient estimates $\hat{\beta }$ are expressed as $1.10^{\hat{\beta }}$ for IgG _pre-challenge_ antibody titers and as $e^{\hat{\beta }}$ for all other parameters.

### Antibody responses following PPV immunization versus responses to low-dose challenge

We next compared children’s antibody titers 1 month after challenge with a low dose of PPV23 with their titers at 10 months of age, 1 month after PPV23 vaccination.

**Fig 4:**
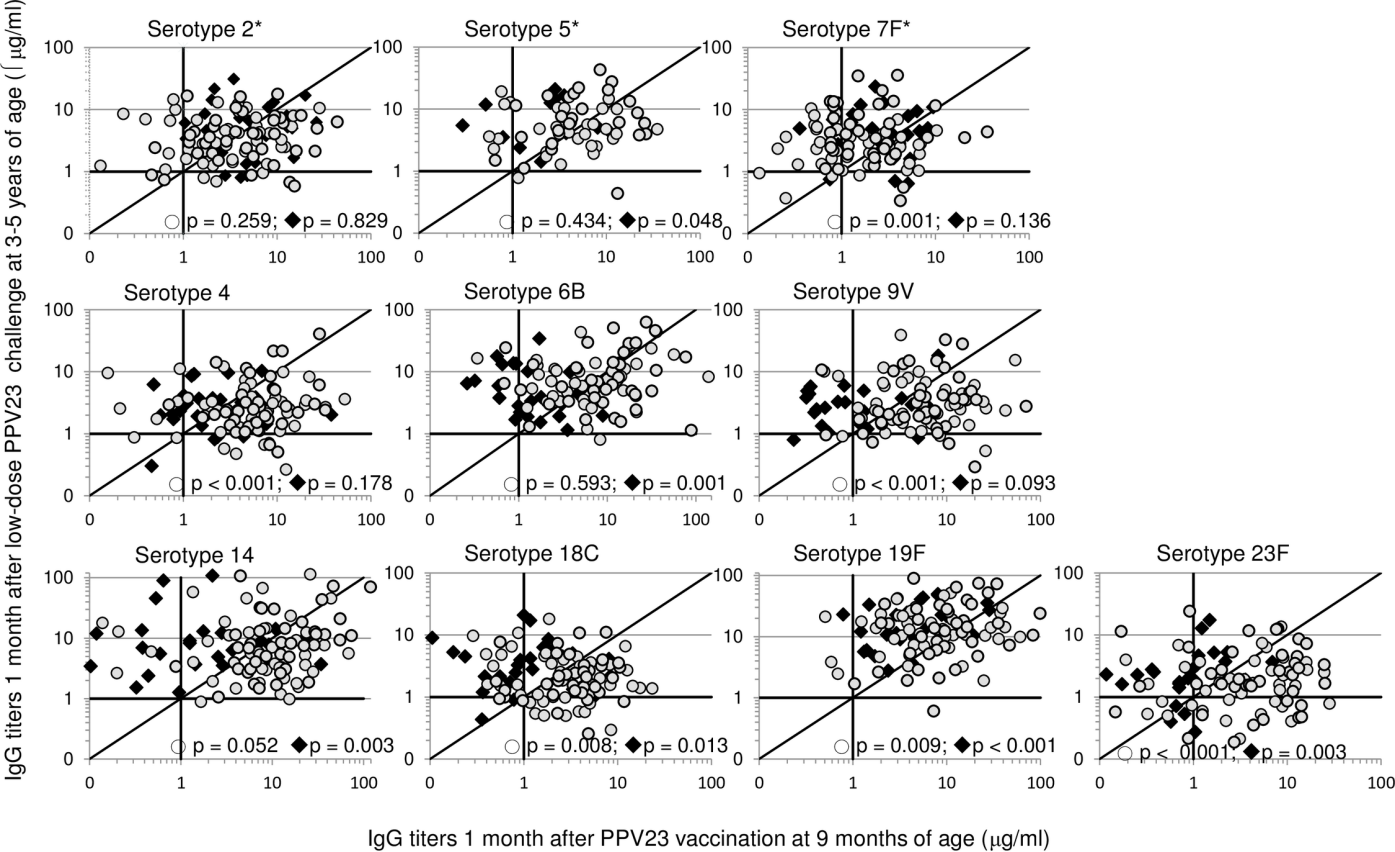
Individual pneumococcal serotype-specific IgG antibody titers following PPV23 vaccination at 9 months versus low-dose challenge at 3–5 years of age. Graphs present scatterplots of pneumococcal serotype-specific IgG antibody titers measured in individual children 1 month after PPV23 vaccination (vaccinated at 9 months of age) (x axis) and 1 month after challenge with a low dose of PPV23 at 3–5 years of age (y axis), in those who had received 3 doses of PCV7 before the PPV23 vaccine (primed; grey circles), or only received PPV23 (unprimed; black diamonds). Serotypes with an asterisk (*) are included in PPV23 only, while the other serotypes are included in both PCV7 and PPV23. The p-values included in the graphs correspond with non-parametric analysis of paired responses within the treatment groups (Wilcoxon Signed Rank Test).

As reported previously [13] and illustrated in Fig 4, antibody titers against PCV7/PPV23 serotypes were higher in the PCV7-primed group than in the unprimed group one month after PPV23 vaccination in infancy. Following a low dose PPV23 challenge at 3–5 years of age, IgG titers were higher than (for serotypes 5, 6B, 14, 18C, 19F and 23F) or similar to (for serotypes 2, 4, 7F and 9V) titers one month after PPV23 vaccination in infancy in most children vaccinated with PPV23 only (Fig 4), while in the PCV7-primed group most children had higher titers against serotypes 7F and 19F, similar titers against serotypes 2, 5, 6B and 14, and lower titers against serotypes 4, 9V, 18C and 23F.

### Memory B-cells

**Fig 5:**
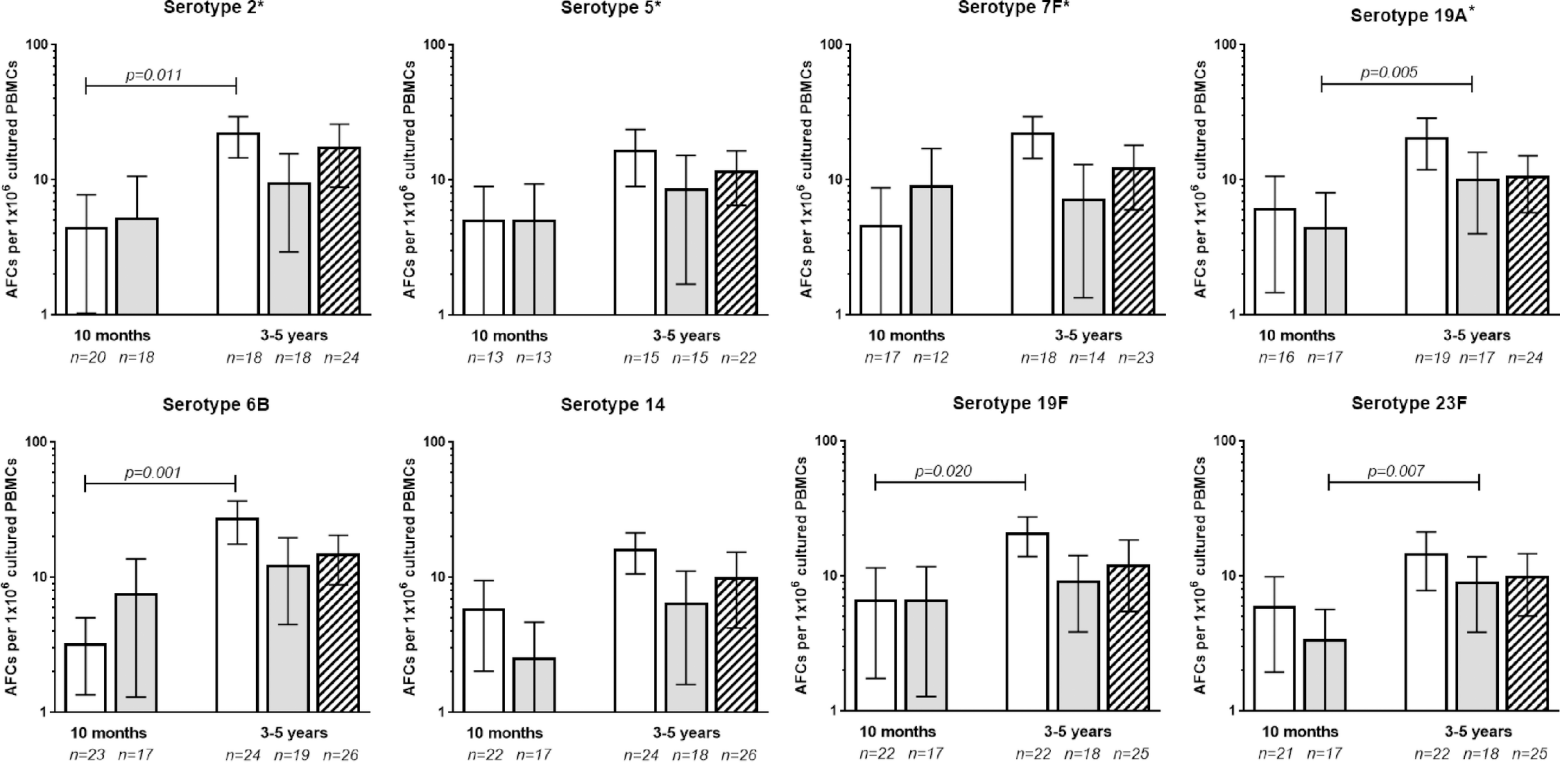
Pneumococcal serotype-specific memory B-cell responses. Serotype-specific Antibody Forming Cells (AFCs) per 1x10^6^ cultured PBMCs were measured at 10 months of age (1 month after PPV23 vaccination) and pre-challenge at 3–5 years of age in a subset of children vaccinated with PCV7 and PPV23 (white bars) or PPV23-only (grey bars), and pre-challenge at 3–5 years of age in a subset of children not vaccinated with any pneumococcal vaccines (striped pattern). Serotypes with an asterisk (*) are included in PPV23 only, while other serotypes are included in both PCV7 and PPV23. Data are presented as means and 95% confidence intervals. No significant differences were found between groups at 10 months or 3–5 years of age (tested using Mann-Whitney U test). Differences in responses at 10 months versus 3–5 years of age within a group were tested using Wilcoxon Signed Rank Test, and where significant differences were found this has been indicated in the graph.

Serotype-specific memory B-cells were assessed for a subset of children at 10 months of age and before challenge at 3–5 years of age. As illustrated in Fig 5, there were no differences in the relative numbers of memory B-cells at 3–5 years of age between children who had never received any pneumococcal vaccines, those who had received PCV7 and PPV23 in the first year of life, or those who had received PPV23 alone. Also, at 10 months of age, one month after PPV23 vaccination, there was no difference in the frequency of serotype-specific memory B cells between children who had been vaccinated with PCV7 or not.

Overall the relative number of memory B-cells remained the same or increased between 10 months and 3–5 years of age, although the increase was only significant for serotype 2 in both groups, and for serotypes 6B and 19F in the group vaccinated with PCV7 and PPV23.

## Discussion

It has been suggested that long-term protection against encapsulated bacteria requires sustained antibody levels in addition to persistence of memory B-cells [19]. How long-term production of antibodies is maintained in humans is not clear, but one suggestion is a continuous turn-over of memory B-cells to replace plasma cells. We found that, regardless of prior pneumococcal vaccination, levels of circulating IgG antibodies against PCV7 and selected PPV23 serotypes were high in all studied 3-5-year-old Papua New Guinean children, with nearly all children having titers ≥0.35 μg/ml and the majority having titers of ≥1.0 μg/ml. Accordingly, numbers of circulating serotype-specific memory B-cells were similar in prior pneumococcal vaccine recipients and control children. At the time of our study, no PCVs had yet been introduced in PNG. We have previously reported that PCV7 vaccination in early infancy has limited impact on overall and PCV7-specific pneumococcal carriage [3]. Pneumococcal carriage in the vaccinated and unvaccinated population is therefore expected to be high. Indeed, we found that 80% of the 3-5-year-old children carried pneumococci at the time of the challenge, 18–35% of which were PCV7 serotypes, with no differences between groups [20]. High natural pneumococcal exposure is likely to have induced and maintained pneumococcal immune responses in our study population, with no evidence that prior pneumococcal vaccination contributed to higher antibody responses at 3–5 years of age. This is in line with findings of a study in Native American children reporting that PCV7 immunization in infancy did not result in persisting immune memory 4 years post-vaccination [21]; in contrast to our study, in this community PCV7 introduction had resulted in a significant reduction of circulating PCV7 serotypes and antibody titers were consequently low in both PCV7-vaccinated and unvaccinated children.

It is important to note that most pneumococcal disease and deaths occur in the first two years of life. In line with other studies, in our neonatal PCV7 trial we have shown that three doses of PCV7 administered in early infancy followed by PPV23 at 9 months of age induce protective antibody responses against vaccine serotypes in most children for at least the first 18 months of life [13]. While our current study indicates that these pneumococcal vaccinations do not result in higher levels of protection at 3–5 years of age compared to children who have acquired natural immunity, vaccinated children will have been better protected against pneumococcal infections than unvaccinated children at least during the first two years of life when the burden of pneumococcal disease is the highest and protection is most critical.

In this study, we measured anamnestic responses by challenging study subjects with a low dose of PPV23. While this may not induce a similar immune response as a pneumococcal infection, its advantage is that immune responses to 23 different pneumococcal serotypes can be measured. In line with our conclusion that PCVs do not induce long-lasting immune memory, we found that responses to challenge with PPV23 were similar in PCV7-vaccinated and unvaccinated children, with the majority showing similar or higher antibody titers post-challenge to all tested serotypes compared to pre-challenge titers. Since pre-challenge antibody titers were high in most children, increases in antibody responses were modest, and were inversely related to pre-challenge titers. This phenomenon that the relative increase in antibody titers in response to a booster (or challenge) is lower when pre-existing titers are high, has been reported for other vaccines, including meningococcal conjugate vaccine [19], a candidate *Escherichia coli* conjugate vaccine [22], and tetanus and diphtheria vaccines [23, 24]. Although the underlying mechanism is unclear, a feedback mechanism appears to be in place that prevents unlimited activation of B cells above a certain antibody level, which is consistent with other regulatory mechanisms built into the immune system. What this means from a clinical perspective is unclear, and for our study children we can only hypothesize that their high circulating antibody titers will protect them against invasive pneumococcal disease.

PCVs are effective in preventing the most serious forms of vaccine serotype pneumococcal disease in young children [25]; however, the number of serotypes covered by current PCVs is limited, leaving young infants in highly endemic settings at risk of infections by non-PCV serotypes [4, 26]. Administration of one dose of PPV23 after priming with three doses of one of the currently available PCVs in early infancy, may provide young children in high-risk settings with additional protection against infections due to serotypes not included in the used PCV, as has been demonstrated in earlier studies in PNG [9–11]. This would be particularly important in the first 2 years of life when the burden of severe pneumococcal infections is highest. However, there are concerns that polysaccharide vaccines may lead to immune hypo-responsiveness by triggering the activation and recruitment of memory B-cells to differentiate into antibody-secreting plasma cells and hence resulting in depletion of the memory B-cell compartment [27, 28].

A study in Fiji reported that children who received 0 to 3 doses of PCV7 and were randomized to receive PPV23 at 12 months, failed to further boost their antibody responses when challenged 5 months later with a low dose of PPV23 [29]. While responses after the challenge were not lower but similar to responses 1 month after PPV23 immunization, children who had not received PPV23 showed higher antibody levels to PCV7 and additional serotypes in response to the challenge. While this may indicate that PPV23 immunization following PCV priming may affect anamnestic responses in the short term, it should be noted that children maintained high protective antibody titers that often exceeded 1.0 μg/ml after the challenge. Moreover, when these children were followed five years later and given a booster vaccination with PCV13, there was no immunologic evidence of hypo-responsiveness [30]. A study conducted in Iceland reported that children primed with 2 or 3 doses of PCV9 before the age of 1 year, followed by a dose of PCV9 or PPV23 at 12 months of age, produced lower antibody responses to PCV13 booster vaccination at 7.5 years of age if they had received PPV23 than if they had received PCV9 at 12 months of age [31]. Whereas the investigators concluded that PPV23 after priming with PCVs had a detrimental impact on the ability of children to respond to subsequent vaccination with PCV13, the observed difference in the immune responses to PCV13 at 7.5 years of age could also be the result of the additional dose of PCV9 at 12 months enhancing immune memory in the non-PPV23 group. Of note, PCV13 booster vaccination was immunogenic in all children, regardless of prior vaccination with PCV-only or PCV followed by PPV23, and most children had antibody titers ≥ 0.35 μg/ml against all tested serotypes at 7.5 years of age.

In our study we found that most children vaccinated with PPV23-only produced higher antibody titers in response to the low dose PPV23 challenge at 3–5 years of age than to PPV23 vaccination at 9 months of age. This indicates firstly the development of immune memory in the intermediate period as a result of natural exposure; and secondly that PPV23 vaccination without prior PCV priming did not result in long-term hypo-responsiveness. In contrast, in most children vaccinated with PCV7 and PPV23, antibody titers for five PCV7 serotypes were lower after the low-dose PPV23 challenge than after PPV23 immunization at 9 months of age. However, antibody responses against all PCV7 serotypes were significantly higher following the 9-month PPV23 vaccination in the PCV7-primed group than in the non-PCV7 (PPV23 only) group [13]. Questioning as to whether it is biologically plausible to find similarly high antibody responses 2–4 years later in response to 1/5^th^ of the vaccine dose, we believe that the relatively lower response to a low dose PPV23 challenge at 3–5 years of age in the PCV7-primed group does not signify hypo-responsiveness, but relates to the high antibody responses that these children produce in response to PPV23 vaccination at 9 months of age that is unlikely to be matched by a similar or higher response to 1/5^th^ of the normal dose.

Use of pneumococcal vaccines in high-risk settings has rightfully focused on early infant immunization protecting the most susceptible age group against severe infections. Yet, boosting of the immune response with PCVs in later infancy may improve long-term persistence of immune memory as shown by a study in France where children vaccinated with 3 doses of PCV7 or PCV13 in infancy followed by a toddler booster dose produced increased antibody titers to a subsequent dose of PCV13 administered at least 2 years after the toddler dose [32]. However, introduction of an extra dose of PCV may not be achievable in low-income settings. Alternatively, a study in Nepal demonstrated that a two-dose prime with a booster at age 9 months (2+1) compared with a three-dose primary PCV10 schedule (3+0) improved antibody persistence through early childhood without compromising antibody responses in early infancy [33]. While this could be a promising approach that would need further evidence from highly endemic settings, higher valency PCVs or species-wide pneumococcal vaccines are needed to provide broader protection to children in highly endemic settings like PNG [4]. PPV23 in later infancy could provide a certain level of additional protection that children in high-risk settings currently lack. The Fijian and our study show that antibody titers remain high following PPV23 immunization and that there is no evidence for lasting hypo-responsiveness.

A limitation of our study is that our initial neonatal PCV7 trial did not include a study group that did not receive PPV23 at 9 months of age, i.e. was vaccinated with PCV7 only. We are currently analysing data from a second, recently completed open randomised controlled trial in PNG comparing the immunogenicity of 3 doses of PCV10 or PCV13 at 1, 2 and 3 months of age, followed by PPV23 or no PPV23 at 9 months of age (ClinicalTrials.gov identifier NCT01619462). Children were then challenged with a low dose of PPV23 at 2 years of age to assess potential intermediate-term effects of PPV23 on the immune response. This will provide additional information on the immunological effect of PPV23 in children primed with PCVs in a high-risk setting. Another limitation of our study is the lower number of children in the PPV23-only group that could be recruited for the low-dose PPV23 challenge follow-up study; while we cannot identify a reason for the lower recruitment, the relatively low number of children in this group has meant low statistical power for certain analyses such as comparing antibody responses at the different time points.

In summary, we found that in a highly endemic setting like PNG natural pneumococcal exposure leads to the induction of naturally acquired immunity that is as high at age 3–5 years as in children vaccinated in infancy with pneumococcal vaccines. Finally, we found no evidence that immunization with PPV23 after PCV priming in the first year of life results in hypo-responsiveness.

## Supporting information

S1 Table Generalised estimating equation model coefficient estimates of the association of pre-challenge serotype-specific antibody concentrations with PPV23 vaccination status, gender and age prior to challenge. (DOCX)

S2 Table Generalised estimating equation model coefficient estimates of the association between pre-challenge serotype-specific antibody concentrations and PCV7 vaccination status, gender and age prior to challenge for those children vaccinated with PPV23 at 9 months of age. (DOCX)

S1 Text Checklist. (PDF)

S2 Text Protocol. (PDF)

S1 Data ELISA data. (XLS)

## References

[1] O'Brien KL, Wolfson LJ, Watt JP, Henkle E, Deloria-Knoll M, McCall N, et al Burden of disease caused by *Streptococcus pneumoniae* in children younger than 5 years: global estimates. Lancet. 2009;374(9693):893–902. doi: 10.1016/S0140-6736(09)61204-6 19748398 10.1016/S0140-6736(09)61204-6

[2] Whitney CG, Goldblatt D, O'Brien KL. Dosing schedules for pneumococcal conjugate vaccine: considerations for policy makers. Pediatr Infect Dis J. 2014;33 Suppl 2:S172–81. 24336059 10.1097/INF.0000000000000076 PMC3940379

[3] Aho C, Michael A, Yoannes M, Greenhill AR, Jacoby P, Reeder J, et al Limited impact of neonatal or early infant schedules of 7-valent pneumooccal conjugate vaccination on nasopharyngeal carriage of *Streptococcus pneumoniae* in Papua New Guinean children: A randomized controlled trial. Vaccine Reports. 2016;6:36–43. doi: 10.1016/j.vacrep.2016.08.002 28580433 10.1016/j.vacrep.2016.08.002 PMC5446595

[4] Greenhill AR, Phuanukoonnon S, Michael A, Yoannes M, Orami T, Smith H, et al *Streptococcus pneumoniae* and *Haemophilus influenzae* in paediatric meningitis patients at Goroka General Hospital, Papua New Guinea: serotype distribution and antimicrobial susceptibility in the pre-vaccine era. BMC Infect Dis. 2015;15:485 doi: 10.1186/s12879-015-1197-0 26521138 10.1186/s12879-015-1197-0 PMC4628371

[5] Laferriere C. The immunogenicity of pneumococcal polysaccharides in infants and children: a meta-regression. Vaccine. 2011;29(40):6838–47. doi: 10.1016/j.vaccine.2011.07.097 21816198 10.1016/j.vaccine.2011.07.097

[6] Lee LH, Frasch CE, Falk LA, Klein DL, Deal CD. Correlates of immunity for pneumococcal conjugate vaccines. Vaccine. 2003;21(17–18):2190–6. 12706710 10.1016/s0264-410x(03)00025-2

[7] Le Polain De Waroux O, Flasche S, Prieto-Merino D, Goldblatt D, Edmunds WJ. The efficacy and duration of protection of pneumococcal conjugate vaccines against nasopharyngeal carriage: a meta-regression model. Pediatr Infect Dis J. 2015;34(8):858–64. doi: 10.1097/INF.0000000000000717 26075814 10.1097/INF.0000000000000717

[8] Phuanukoonnon S, Reeder J, Pomat W, Van den Biggelaar A, Holt P, Saleu G, et al A neonatal pneumococcal conjugate vaccine trial in Papua New Guinea: study population, methods and operational challenges. PNG Med J 2010 53(3–4):191–206. Available at: http://pngimr.org.pg/png_med_journal/sept-dec%202010%20a%20neonatal%20pneumococcal%20conjugate%20vaccine%20trials%20in%20png.pdf. 23163191

[9] Lehmann D, Marshall TF, Riley ID, Alpers MP. Effect of pneumococcal vaccine on morbidity from acute lower respiratory tract infections in Papua New Guinean children. Ann Trop Paediatr. 1991;11(3):247–57. 1719924 10.1080/02724936.1991.11747510

[10] Riley ID, Lehmann D, Alpers MP, Marshall TF, Gratten H, Smith D. Pneumococcal vaccine prevents death from acute lower-respiratory-tract infections in Papua New Guinean children. Lancet. 1986;2(8512):877–81. 2876325 10.1016/s0140-6736(86)90409-5

[11] Pomat WS, Lehmann D, Sanders RC, Lewis DJ, Wilson J, Rogers S, et al Immunoglobulin G antibody responses to polyvalent pneumococcal vaccine in children in the highlands of Papua New Guinea. Infect Immun. 1994;62(5):1848–53. 8168948 10.1128/iai.62.5.1848-1853.1994 PMC186424

[12] van den Biggelaar AH, Pomat W, Bosco A, Phuanukoonnon S, Devitt CJ, Nadal-Sims MA, et al Pneumococcal conjugate vaccination at birth in a high-risk setting: No evidence for neonatal T-cell tolerance. Vaccine. 2011;29(33):5414–20. doi: 10.1016/j.vaccine.2011.05.065 21645573 10.1016/j.vaccine.2011.05.065 PMC3146700

[13] Pomat WS, van den Biggelaar AH, Phuanukoonnon S, Francis J, Jacoby P, Siba PM, et al Safety and immunogenicity of neonatal pneumococcal conjugate vaccination in Papua New Guinean children: a randomised controlled trial. PLoS One. 2013;8(2):e56698 doi: 10.1371/journal.pone.0056698 23451070 10.1371/journal.pone.0056698 PMC3579820

[14] Siber GR, Chang I, Baker S, Fernsten P, O'Brien KL, Santosham M, et al Estimating the protective concentration of anti-pneumococcal capsular polysaccharide antibodies. Vaccine. 2007;25(19):3816–26. doi: 10.1016/j.vaccine.2007.01.119 17368878 10.1016/j.vaccine.2007.01.119

[15] Clutterbuck EA, Lazarus R, Yu LM, Bowman J, Bateman EA, Diggle L, et al Pneumococcal conjugate and plain polysaccharide vaccines have divergent effects on antigen-specific B cells. J Infect Dis. 2012;205(9):1408–16. doi: 10.1093/infdis/jis212 22457293 10.1093/infdis/jis212 PMC3324398

[16] Fuery A, Richmond PC, Currie AJ. Human infant memory B cell and CD4+ T cell responses to HibMenCY-TT glyco-conjugate vaccine. PLoS One. 2015;10(7):e0133126 doi: 10.1371/journal.pone.0133126 26191794 10.1371/journal.pone.0133126 PMC4507978

[17] R Core Team. R: a language and environment for statistical computing Vienna, Austria: R Foundation for Statistical Computing; 2013.

[18] Carey VJ, Lumley T, Ripley B. GEE: Generalized Estimation Equation solver. 2012. http://CRAN.R-project.org/package=gee. R package version 4.13–18. [p181].

[19] Blanchard Rohner G, Snape MD, Kelly DF, John T, Morant A, Yu LM, et al The magnitude of the antibody and memory B cell responses during priming with a protein-polysaccharide conjugate vaccine in human infants is associated with the persistence of antibody and the intensity of booster response. J Immunol. 2008;180(4):2165–73. 18250423 10.4049/jimmunol.180.4.2165

[20] Yoannes M, Michael A, Saleu G, Opa C, Greenhill A, Siba P, et al. High pneumococcal carriage in 3–5 year old Papua New Guinean children following 9-month Pneumovax23 dose with or without prior 7-valent conjugate vaccine. Eighth International Symposium on Pneumococci and Pneumococcal Diseases (ISPPD8); Iguacu, Brazil, 11–15 March 2012.

[21] Grant LR, Burbidge P, Haston M, Johnson M, Reid R, Santosham M, et al Persistence of IgG antibody following routine infant immunization with the 7-valent pneumococcal conjugate vaccine. Pediatr Infect Dis J. 2015;34(5):e138–42. doi: 10.1097/INF.0000000000000655 25621761 10.1097/INF.0000000000000655

[22] Cross A, Artenstein A, Que J, Fredeking T, Furer E, Sadoff JC, et al Safety and immunogenicity of a polyvalent *Escherichia coli* vaccine in human volunteers. J Infect Dis. 1994;170(4):834–40. 7523536 10.1093/infdis/170.4.834

[23] Danilova E, Shiryayev A, Kristoffersen EK, Sjursen H. Attenuated immune response to tetanus toxoid in young healthy men protected against tetanus. Vaccine. 2005;23(42):4980–3. doi: 10.1016/j.vaccine.2005.05.028 15985319 10.1016/j.vaccine.2005.05.028

[24] Danilova E, Shiryayev A, Skogen V, Kristoffersen EK, Sjursen H. Short-term booster effect of diphtheria toxoid in initially long-term protected individuals. Vaccine. 2005;23(12):1446–50. doi: 10.1016/j.vaccine.2004.09.020 15670879 10.1016/j.vaccine.2004.09.020

[25] Cutts FT, Zaman SM, Enwere G, Jaffar S, Levine OS, Okoko JB, et al Efficacy of nine-valent pneumococcal conjugate vaccine against pneumonia and invasive pneumococcal disease in The Gambia: randomised, double-blind, placebo-controlled trial. Lancet. 2005;365(9465):1139–46. doi: 10.1016/S0140-6736(05)71876-6 15794968 10.1016/S0140-6736(05)71876-6

[26] Saha SK, Hossain B, Islam M, Hasanuzzaman M, Saha S, Hasan M, et al Epidemiology of invasive pneumococcal disease in Bangladeshi children before introduction of pneumococcal conjugate vaccine. Pediatr Infect Dis J. 2016;35(6):655–61. doi: 10.1097/INF.0000000000001037 26658530 10.1097/INF.0000000000001037

[27] Granoff DM, Pollard AJ. Reconsideration of the use of meningococcal polysaccharide vaccine. Pediatr Infect Dis J. 2007;26(8):716–22. doi: 10.1097/INF.0b013e3180cc2c25 17848884 10.1097/INF.0b013e3180cc2c25

[28] Poolman J, Borrow R. Hyporesponsiveness and its clinical implications after vaccination with polysaccharide or glycoconjugate vaccines. Expert Rev Vaccines. 2011;10(3):307–22. doi: 10.1586/erv.11.8 21434799 10.1586/erv.11.8

[29] Russell FM, Carapetis JR, Balloch A, Licciardi PV, Jenney AW, Tikoduadua L, et al Hyporesponsiveness to re-challenge dose following pneumococcal polysaccharide vaccine at 12 months of age, a randomized controlled trial. Vaccine. 2010;28(19):3341–9. doi: 10.1016/j.vaccine.2010.02.087 20206670 10.1016/j.vaccine.2010.02.087 PMC2854305

[30] Licciardi PV, Toh ZQ, Clutterbuck EA, Balloch A, Marimla RA, Tikkanen L, et al No long-term evidence of hyporesponsiveness after use of pneumococcal conjugate vaccine in children previously immunized with pneumococcal polysaccharide vaccine. J Allergy Clin Immunol. 2016;137(6):1772–9. doi: 10.1016/j.jaci.2015.12.1303 26825000 10.1016/j.jaci.2015.12.1303 PMC4899320

[31] Sigurdardottir ST, Center KJ, Davidsdottir K, Arason VA, Hjalmarsson B, Elisdottir R, et al Decreased immune response to pneumococcal conjugate vaccine after 23-valent pneumococcal polysaccharide vaccine in children. Vaccine. 2014;32(3):417–24. doi: 10.1016/j.vaccine.2013.11.029 24300594 10.1016/j.vaccine.2013.11.029

[32] Quinet B, Laudat F, Gurtman A, Patterson S, Sidhu M, Gruber WC, et al Pneumococcal conjugate vaccine-elicited antibody persistence and immunogenicity and safety of 13-valent pneumococcal conjugate vaccine in children previously vaccinated with 4 doses of either 7-valent or 13-valent pneumococcal conjugate vaccine. Pediatr Infect Dis J. 2014;33(10):1065–76. doi: 10.1097/INF.0000000000000459 25093973 10.1097/INF.0000000000000459

[33] Hamaluba M, Kandasamy R, Upreti SR, Subedi GR, Shrestha S, Bhattarai S, et al Comparison of two-dose priming plus 9-month booster with a standard three-dose priming schedule for a ten-valent pneumococcal conjugate vaccine in Nepalese infants: a randomised, controlled, open-label, non-inferiority trial. Lancet Infect Dis. 2015;15(4):405–14. doi: 10.1016/S1473-3099(15)70007-1 25701560 10.1016/S1473-3099(15)70007-1

