# Supplementary material for: Pneumococcal responses are similar in Papua New Guinean children aged 3-5 years vaccinated in infancy with pneumococcal polysaccharide vaccine with or without prior pneumococcal conjugate vaccine, or without pneumococcal vaccination
Source: PLoS One. 2017 Oct 13;12(10):e0185877. doi: 10.1371/journal.pone.0185877 (PMC5640225; doi:10.1371/journal.pone.0185877)
Supplement: S1 Table — (DOCX) [file pone.0185877.s001.docx]

**S1 Table.** Generalised estimating equation model (gee) coefficient estimates of the association of (log-transformed) pre-challenge serotype-specific antibody concentrations with PPV23 vaccination status, gender and age prior to challenge.

|  | **S2** | **S4** | **S5** | **S6B** | **S7F** | **S9V** | **S14** | **S18C** | **S19F** | **S23F** |
| --- | --- | --- | --- | --- | --- | --- | --- | --- | --- | --- |
|  | $\boldsymbol{e}^{\hat{\boldsymbol{\beta}}}$ **(95% CI)** | $\boldsymbol{e}^{\hat{\boldsymbol{\beta}}}$ **(95% CI)** | $\boldsymbol{e}^{\hat{\boldsymbol{\beta}}}$ **(95% CI)** | $\boldsymbol{e}^{\hat{\boldsymbol{\beta}}}$ **(95% CI)** | $\boldsymbol{e}^{\hat{\boldsymbol{\beta}}}$ **(95% CI)** | $\boldsymbol{e}^{\hat{\boldsymbol{\beta}}}$ **(95% CI)** | $\boldsymbol{e}^{\hat{\boldsymbol{\beta}}}$ **(95% CI)** | $\boldsymbol{e}^{\hat{\boldsymbol{\beta}}}$ **(95% CI)** | $\boldsymbol{e}^{\hat{\boldsymbol{\beta}}}$ **(95% CI)** | $\boldsymbol{e}^{\hat{\boldsymbol{\beta}}}$ **(95% CI)** |
| **PPV 23** |  |  |  |  |  |  |  |  |  |  |
| No | 1 | 1 | 1 | 1 | 1 | 1 | 1 | 1 | 1 | 1 |
| Yes | 1.03 (0.80-1.33) | 1.11 (0.86-1.45) | 0.98 (0.76-1.27) | 1.12 (0.88-1.44) | 0.86 (0.65-1.14) | 1.20 (0.93-1.56) | 1.12 (0.86-1.47) | 1.14 (0.89-1.46) | 1.01 (0.79-1.28) | 0.88 (0.67-1.14) |
| **Gender** |  |  |  |  |  |  |  |  |  |  |
| Female | 1 | 1 | 1 | 1 | 1 | 1 | 1 | 1 | 1 | 1 |
| Male | 0.83 (0.65-1.06) | 0.91 (0.70-1.19) | 0.86 (0.67-1.11) | 0.86 (0.68-1.10) | 0.91 (0.69-1.19) | 0.78 (0.60-1.00) | 0.82 0.63-1.06) | 0.82 (0.64-1.04) | 0.84 (0.66-1.07) | 0.88 (0.68-1.15) |
| **Age (years)** | 1.21 (1.04-1.40) | 1.29 (1.08-1.54) | 1.39 (1.19-1.62) | 1.39 (1.19-1.62) | 1.43 1.21-1.68) | 1.38 1.18-1.62) | 1.32 (1.12-1.55) | 1.47 1.25-1.72) | 1.26 1.08-1.47) | 1.37 1.17-1.61) |

This analysis shows that vaccination with PPV23 at 9 months of age was not significantly associated with pre-challenge serotype-specific antibody concentrations. Increasing age is associated with increasing pre-challenge serotype-specific antibody concentrations. For a 1 year increase in age, geometric mean pre-challenge serotype-specific antibody concentrations increase anywhere between 21% [95% CI: 4-40%; Serotype 2] and 47% [95% CI: 25-72%; Serotype 18C].
