# Supplementary material for: Pneumococcal responses are similar in Papua New Guinean children aged 3-5 years vaccinated in infancy with pneumococcal polysaccharide vaccine with or without prior pneumococcal conjugate vaccine, or without pneumococcal vaccination
Source: PLoS One. 2017 Oct 13;12(10):e0185877. doi: 10.1371/journal.pone.0185877 (PMC5640225; doi:10.1371/journal.pone.0185877)
Supplement: S2 Table — (DOCX) [file pone.0185877.s002.docx]

**S2 Table.** Generalised estimating equation model (gee) coefficient estimates of the association between (log-transformed) pre-challenge serotype-specific antibody concentrations and PCV7 vaccination status, gender and age prior to challenge for those children vaccinated with PPV23 at 9 months of age.

|  | **S2** | **S4** | **S5** | **S6B** | **S7F** | **S9V** | **S14** | **S18C** | **S19F** | **S23F** |
| --- | --- | --- | --- | --- | --- | --- | --- | --- | --- | --- |
|  | $\boldsymbol{e}^{\hat{\boldsymbol{\beta}}}$  **(95% CI)** | $\boldsymbol{e}^{\hat{\boldsymbol{\beta}}}$  **(95% CI)** | $\boldsymbol{e}^{\hat{\boldsymbol{\beta}}}$  **(95% CI)** | $\boldsymbol{e}^{\hat{\boldsymbol{\beta}}}$  **(95% CI)** | $\boldsymbol{e}^{\hat{\boldsymbol{\beta}}}$  **(95% CI)** | $\boldsymbol{e}^{\hat{\boldsymbol{\beta}}}$  **(95% CI)** | $\boldsymbol{e}^{\hat{\boldsymbol{\beta}}}$  **(95% CI)** | $\boldsymbol{e}^{\hat{\boldsymbol{\beta}}}$  **(95% CI)** | $\boldsymbol{e}^{\hat{\boldsymbol{\beta}}}$  **(95% CI)** | $\boldsymbol{e}^{\hat{\boldsymbol{\beta}}}$  **(95% CI)** |
| **PCV7** |  |  |  |  |  |  |  |  |  |  |
| No | 1 | 1 | 1 | 1 | 1 | 1 | 1 | 1 | 1 | 1 |
| Yes | 0.93  (0.64-1.35) | 1.06  (0.70-1.61) | 0.87  (0.60-1.26) | 1.20  (0.82-1.75) | 0.75  (0.51-1.11) | 0.96  (0.65-1.42) | 1.00  (0.68-1.48) | 1.01  (0.70-1.46) | 0.91  (0.64-1.28) | 0.90  (0.60-1.36) |
| **Gender** |  |  |  |  |  |  |  |  |  |  |
| Female | 1 | 1 | 1 | 1 | 1 | 1 | 1 | 1 | 1 | 1 |
| Male | 0.93  (0.65-1.32) | 1.01  (0.73-1.39) | 0.96  (0.68-1.35) | 0.96  (0.71-1.30) | 0.88  (0.63-1.25) | 0.92  (0.67-1.27) | 0.80  (0.57-1.12) | 0.94  (0.70-1.26) | 0.86  (0.64-1.17) | 0.92  (0.65-1.29) |
| **Age (years)** | 1.17  (0.90-1.53) | 1.34  (0.99-1.82) | 1.51  (1.15-1.97) | 1.42  (1.11-1.81) | 1.65  (1.25-2.16) | 1.51  (1.17-1.95) | 1.51  (1.17-1.96) | 1.76  (1.38-2.25) | 1.40  (1.12-1.76) | 1.66  (1.28-2.17) |

This analysis shows that prior vaccination with PCV7 was not significantly associated with pre-challenge serotype-specific antibody titers amongst children vaccinated with PPV23 at 9 months of age. Increasing age is associated with increasing pre-challenge serotype-specific antibody concentrations for most serotypes. For a 1 year increase in age, geometric mean pre-challenge serotype-specific antibody concentrations increase anywhere between 40% [95% CI: 12-76%; Serotype 19F] and 76% [95% CI: 38-125%; Serotype 18C].
