# Supplementary material for: Pneumococcal responses are similar in Papua New Guinean children aged 3-5 years vaccinated in infancy with pneumococcal polysaccharide vaccine with or without prior pneumococcal conjugate vaccine, or without pneumococcal vaccination
Source: PLoS One. 2017 Oct 13;12(10):e0185877. doi: 10.1371/journal.pone.0185877 (PMC5640225; doi:10.1371/journal.pone.0185877)
Supplement: S2 Text — (PDF) [file pone.0185877.s004.pdf]

Protocol Full title:
Investigation of Serotype-specific Antibody persistence and B-cell memory at age 3 - 5 years following 23-valent Pneumococcal Polysaccharide vaccine at age 9 months in Papua New Guinean children previously primed with 7-valent pneumococcal conjugate vaccine

Investigators
A/Professor Peter Richmond, University of Western Australia School of Paediatrics & Child health
Dr William Pomat, Papua New Guinea Institute of Medical Research
A/Professor Deborah Lehmann, Telethon Institute for Child Health Research
Professor Peter Siba, Papua New Guinea Institute of Medical Research
Dr Anita van den Biggelaar, Telethon Institute for Child Health Research
Dr Andrew Greenhill, Papua New Guinea Institute of Medical Research
Mr Peter Jacoby, Telethon Institute for Child Health Research

1.1 Investigational Product
23-valent pneumococcal polysaccharide vaccine containing 25µg polysaccharide in 0.5mL of each of the following serotypes: 1, 2, 3, 4, 5, 6B, 7F, 8, 9N, 9V, 10A,11A,12F, 14, 15B, 17F, 18C, 19A, 19F, 20, 22F, 23F, 33F

1.2 Background
Pneumococcal disease: a major global health problem of young children
It is estimated that 2.1 million children die annually from pneumonia – 'more than from HIV, tuberculosis and malaria combined'[1] – the majority in the developing world; *Streptococcus pneumoniae* is responsible for approximately one million of these deaths.[2] The pneumococcus is also a leading cause of meningitis, bacteraemia and otitis media (OM). Young infants are at particularly high risk of pneumococcal disease. Interventions to prevent this mortality and morbidity in infants are urgently needed.[2]

Burden of pneumococcal disease in Papua New Guinean children: Pneumonia is the most common reason for childhood hospitalisation and mortality in PNG. In the Asaro Valley, Eastern Highlands Province (EHP), where we will conduct the study, mortality rate for acute lower respiratory tract infection (ALRI) in infants is 25/1000 live births/year.[3] Pneumococcal infection accounts for 46% of bacteraemic pneumonia[4] and ~45% of bacterial meningitis.[5] This risk of pneumonia and IPD has remained unchanged over the last 2 decades. In 2004, we initiated a study of neonatal immunisation with 7V-PCV in the Asaro Valley. In the 109 infants in the control arm who did not receive PCV and were followed up to 18 months of age, there have been 66 episodes of moderate or severe ALRI, 3 episodes of IPD associated with pneumonia (serotypes 18C, 2 & 33); this equates to rates of 3,000/10^5/annum). There were also 2 ALRI-associated deaths.[6] The rates of infant morbidity and mortality due to ALRI and IPD in PNG are among the highest documented in the world and cost-effective interventions are needed to decrease this disease burden.

The role of pneumococcal polysaccharide vaccine (PPV) in PNG children
Studies on the use of PPV in young PNG children at 6 months to 5 years of age demonstrated that PPV prevents death from ALRI (efficacy 59% and 50% in children aged <5 and <2 years, respectively) with 19% reduction in total mortality in the vaccinated group[7] similar to that achieved by the use of 9V-PCV in the Gambia.[8] PPV was less effective in children in industrialised countries[9] but the demonstrated efficacy in PNG may be explained by the high incidence of severe disease and mortality and increased prevalence of immunogenic PPV serotypes causing disease in PNG infants. Serotype-dependent maturation of antibody responses support efficacy data, with 2-fold rises in IgG titres one month post-PPV by 9 months of age for serotypes 2 ,7F and 23F, but only after 18 months for PCV serotypes 6B, 14 and 19F.[10] Therefore a PPV booster in children primed with PCV for these less immunogenic serotypes should provide better protection. Our

findings in the current study support this approach with significantly higher booster responses to PPV in children primed with 7V-PCV under the infant or neonatal schedule. This confirms the induction of immunologic memory by PCV and provides infants with higher antibody levels to at least 18 months of age for most serotypes in comparison to PPV alone. In addition excellent responses were seen to the important non-PCV serotypes 2, 5 7F, as 94.4%, 98.5% and 90% of children achieved IgG titres of >0.35ug/mL at 10 months of age post-PPV, with evidence for good persistence to 18 months of age (Pomat unpublished).

Concerns have been raised regarding potential immunological hypo-responsiveness due to depletion of memory B-cells following PPV in young children even in children primed with PCV.[11] A recent study in Fijian children found that a challenge dose (1/5th usual dose; 0.1mL) of PPV 6 months following PPV at 12 months of age in children primed with 1, 2 or 3 doses of PCV in infancy, resulted in relatively smaller increases in serotype-specific antibodies compared to those children who did not receive PPV at 12 months of age (Mulholland personal communication). It should be noted however that the pre-existing antibody levels prior to the challenge dose at 18 months were higher in the children who had received the PPV at 12 months of age, indicating that they were likely to have protective antibodies in the interval period. The duration of antibody persistence and protection from IPD is uncertain. Of further note, pneumococcal carriage rates are lower in Fijian children than in PNG children which may affect the rate of generation of serotype-specific memory B-cells that are likely to be important in long-term protection against pneumococcal disease (in addition to the generation of serotype-independent immunity to pneumococcal surface proteins).[12, 13] The relevance of the Fijian study regarding the use of PPV in other settings (in particular those where mortality and carriage rates are higher) is uncertain. However the persistence of protective antibodies achieved in PNG infants over the period of highest IPD risk and the results of the PNG efficacy studies suggest that hypo-responsiveness is less likely to be a clinically important issue in PNG children, but should be urgently evaluated. Quantification of circulating serotype-specific memory B-cells following pre-& post-PPV challenge will also help inform this debate.

We will therefore use an existing cohort of PNG infants who have been enrolled in a study of neonatal immunisation study with 7-valent PCV and have all received a dose of PPV at approximately 9 months of age and compare their response to a challenge dose of PPV (1/5th usual dose or 0.1mL) intramuscularly at 3-5 years of age with responses in a group of age- and sex-matched controls living in the same area who have not received any pneumococcal vaccines.

1.3 Potential risks and benefits

All children will receive 0.1mL dose of 23 valent PPV as a single intramuscular dose into the deltoid muscle. In the previous study of this dose of PPV in PNG infants, the vaccine was well tolerated and elicited antibody responses that would be expected to be protective against invasive pneumococcal disease. Local reactions (erythema/ swelling/ injection site pain) and mild systemic reactions (fever, headache, irritability) may occur in a minority of subjects. It would be expected that the reduced dose would be less likely to elicit systemic and local reactions compared to the full 0.5mL dose of PPV which was well tolerated in the previous study at 9 months of age (1% of participants had a local reaction and 6.6% had any systemic symptom following PPV co-administered with measles vaccine).

All infants will have their immunisation status checked, and any over-due vaccines will be administered. Participants and their siblings will be treated for any illness present at the time of study visits.

1.4 Treatment regime and justification

The single 0.1mL dose of PPV has been chosen to be a more stringent test of the presence of immunologic memory to capsular polysaccharides than giving a full dose that all children at 3-4 years of age would be expected to mount a robust antibody response to. Given that this dose of PPV

has been found to be immunogenic in naïve Fiji children at 18 months of age, it would be expected that PNG children should respond if hyporesponsiveness is not present.

1.5 Trial Conduct

The trial will be conducted in accordance with this protocol and in compliance with the ICH principles of Good Clinical Practice (GCP) and the PNGIMR and NHMRC guidelines for clinical trials.

1.6 Study population

Papua New Guinean children from the Asaro valley aged 3 to 5 years of age who have previously received 23-valent pneumococcal polysaccharide vaccine as part of the neonatal pneumococcal conjugate vaccine study or unvaccinated children living in the same area.

2. Aims

We aim to determine whether 23-valent pneumococcal polysaccharide vaccine given at 9 months of age:

1) provides enhanced persistence of antibody levels associated with protection from invasive disease at 3 to 4 years of age compared to unvaccinated controls
2) has an impact on the development of serotype -specific B-cell memory at 3 to 5 years of age
3) enhances antibody persistence and B-cell memory for those serotypes included in 7vPCV among children who received 7vPCV in early infancy

3. Study Design

3.1 Study endpoints

3.1.1 Primary objectives

1. Determine the serotype-specific serum IgG antibody geometric mean concentrations (GMC) pre- and 1-month post-immunisation with 0.1mL PPV challenge dose at 3-5 years of age for serotypes contained in 7V- PCV (4, 6B, 9V, 14, 18C, 19F, 23F) and for PPV serotypes 1, 2, 3, 5, 7F, 19A.
2. Determine the proportion of children with serotype-specific serum IgG antibody levels ≥0.35 and ≥1.0ug/mL pre- and post- PPV challenge dose for the above serotypes
3. Determine the geometric mean opsonophagocytic titre (GMT) and the proportion of children with serum opsonophagocytic titres ≥ 8 pre- and post- PPV challenge dose for the above serotypes

3.1.2 Exploratory objectives

1. Measure the number of circulating serotype-specific memory B-cells for serotypes 2, 6B, 7F, 14 and 19F post-primary and pre- & post- booster immunisation.
2. Determine upper respiratory tract pneumococcal serotype-specific carriage rates pre- and 1 month post-immunisation with 0.1mL PPV challenge dose.
3. Determine serum IgG and circulating T-cell responses to pneumococcal proteins PspA, CbpA, and pneumolysin at 3-5 years of age.
4. Measure T-cell responses to PCV carrier protein CRM_{197} at 3-5 years of age.
5. Compare hospitalisation rates for ALRI between 9 and 48 months of age between children who have received previous PPV (with or without prior priming with 7vPCV) and unvaccinated controls.

3.2 Trial design

This study will be an observational controlled study design consisting of 4 Groups:
Group 1: Children receiving 7VPCV at 0-1-2 months of age and PPV at 9 months of age

Group 2: Children receiving 7VPCV at 1-2-3 months of age and PPV at 9 months of age
Group 3: Children who only received PPV at 9 months of age
Group 4: Children who have not received any previous pneumococcal vaccine

Randomisation and blinding
There will be no randomisation in this study as all subjects will receive the same vaccine and dose. Controls will be chosen from the same village or urban area as children participating in the original PCV study and age-matched in order to minimise bias. Laboratory analyses of samples will be performed by staff blinded to group allocation.

3.3 Trial treatment
All children will receive a single 0.1mL intramuscular injection of commercially available Pneumovax23 ™ (distributed in Australia by CSL Ltd) into the deltoid muscle of the arm following parental informed consent, enrolment and collection of the initial blood sample and pernasal swab.

3.4 Duration of participation
Children will be followed up 1 month post vaccination for specimen collection and any serious adverse events documented or severe vaccine-related adverse events documented. Parents will be asked for consent to be contacted for any future follow-up studies depending on results of the current study.

3.5 Stopping rules
Subjects will be discontinued in the study if subjects develop one of the exclusion criteria or withdrawal of parental consent or migration from the study area. The study will halted if more than 10% of participants develop severe local or systemic symptoms following vaccination pending review by ethics committee and an independent data safety monitor.

3.6 Vaccine Accountability
Vaccine will be stored at the PNG Institute for Medical Research and all vaccines given will be documented on a vaccine register

3.7 Randomisation codes maintenance
Not applicable

3.8 Data identification
Data will be entered directly onto a paper case report form and stored at PNG IMR. De-identified data will be entered onto a password-protected database hosted at the PNGIMR. De-identified data may be sent to the Telethon Institute for Child health Research or University of WA School of Paediaitrics and Child Health for analysis.

4. Subject Eligibility and withdrawal

Inclusion and exclusion criteria are specified below:

4.1 Inclusion criteria
1. PNG Infants aged 3 to 5 yrs of age who participated in previous PNG Neonatal PCV study and received PPV between 9 and 12 months of age, or age- and sex-matched controls who live in the same villages or urban area
2. For previous participants in the PNG Neonatal PCV study who were randomized to receive PCV (in either a 0-1-2 or 1-2-3-month schedule), they must have received 3 doses of PCV according to protocol for that study.
3. Informed parental/guardian consent

4.2 Exclusion criteria

1. Known HIV infection or other immunosuppressive condition or treatment.
2. Prior receipt of 2 doses of pneumococcal polysaccharide vaccine

4.3 Withdrawal Criteria

1. Subjects will be withdrawn if they are diagnosed with HIV infection or another immunodeficiency or are treated with immunosuppressive treatment following vaccination and before the follow-up blood sample.
2. Withdrawal of parental consent
3. Receipt of a pneumococcal vaccine following vaccination and prior to post-vaccination blood samples.

5. The treatment(s) to be administered, including the name(s) of all the product(s), the dose(s), the dosing schedule(s), the route/mode(s) of administration, and the treatment period(s), including the follow-up period(s) for subjects for each investigational product treatment/trial treatment group/arm of the trial.

5.1 Investigational Product administration

Following informed parental consent, children will undergo a brief physical assessment to ensure they are well, afebrile and able to be vaccinated safely. They will then have collection of pre-vaccination blood samples and per nasal swabs. All children will then receive a single intramuscular injection of 0.1mL of 23-valent pneumococcal polysaccharide vaccine (Pneumovax™) into the deltoid muscle of the upper arm. This equates to 5µg of polysaccharide of each of the following serotypes: 1, 2, 3, 4, 5, 6B, 7F, 8, 9N, 9V, 10A,11A,12F, 14, 15B, 17F, 18C, 19A, 19F, 20, 22F, 23F, 33F. Children will be observed for 30 minutes following vaccination to ensure no immediate adverse reactions.

5.2 Specimen collection

This study will involve collection of 2 blood samples of 5-9 mL at each visit. Nurses at PNGIMR have extensive experience in venepuncture for blood culture and immunogenicity studies in young children.[14]

5.2.1 Serum (2-4ml) collected pre- & post-PPV challenge dose will be used for measurement of serotype-specific antibody persistence at 3-4 years of age and response to PPV challenge dose.

5.2.2 Heparinised blood (3-5ml) will be collected for cellular immune studies to quantify numbers of circulating memory B-cells pre- and examine persistence of T-cell memory to vaccine antigens following neonatal or early infant 7vPCV where sufficient sample exists. In our neonatal PCV study sufficient blood was collected at >90% of visits for serum and at 70% for peripheral blood mononuclear cells (PBMCs).[15]

5.2.3 Pernasal swabs (PNS) will be collected to determine the effect of previous PCV and PPV on long-term pneumococcal carriage and the impact of booster immunisation with a PPV challenge dose as an indirect assessment of antibody functionality. Swab collection, transport and storage in skim milk-tryptase-glucose-glycerin broth (STGGB) will employ a standardised methodology which has proven to be very effective in our studies in PNG[16-18] and Australia.[19]

5.2 Medication(s)/treatment(s) permitted (including rescue medication) and not permitted before and/or during the trial.

Any medication is permitted based on clinical indication

5.3 Procedures for monitoring subject compliance.

Not applicable as subjects will only have one vaccination administered by study staff and all samples will be collected by study staff

6. Assessment of Efficacy (Study outcomes)

6.1 Specification of the efficacy parameters:

6.1.1 The primary study outcomes will be

a) serotype-specific IgG antibody geometric mean concentrations (ug/mL) pre- and post-0.1mL dose of 23 valent PPV immunisation at 3-4 years of age for the following serotypes 1, 2, 3, 4, 5, 6B, 7F, 9V, 14, 18C, 19A, 19F and 23F

b) the proportion of children with serotype-specific IgG antibody concentrations ≥ 1.0ug/mL pre- and post-0.1mL dose of 23 valent PPV immunisation at 3-4 years of age for the following serotypes 1, 2, 3, 4, 5, 6B, 7F, 9V, 14, 18C, 19A, 19F and 23F

6.2 Methods and timing for assessing, recording, and analysing of efficacy parameters**:**

Laboratory methods

All laboratory staff will be blinded to subject treatment group.

6.2.1 Pneumococcal serology: The primary study outcomes will be serotype-specific IgG antibody levels pre- and post-PPV immunisation.

Serological testing of serum will be performed in the established immunology laboratory at PNGIMR where the assay has been optimized and provided high quality reliable data in our recent 7V-PCV trial. Serotype-specific Pnc IgG serum antibodies will be measured by the standardised WHO enzyme-linked immunosorbent assay (ELISA) as previously described [10] modified with pre-incubation of unknown sera with cell-wall polysaccharide and CPS 22F to improve assay specificity as recommended.[20] The sera will be assayed for IgG for serotypes contained in 7-valent PPV (4, 6B, 9V, 14, 18C, 19F, 23F) and for PPV serotypes 1, 2, 3, 5, 7F, 19A.

6.2.2 *Serotype-specific opsonophagocytic titres*

Serotype-specific opsonophagocytic titres will be measured pre- and post- PPV immunisation for serotypes contained in 7-valent PPV (4, 6B, 9V, 14, 18C, 19F, 23F) and for PPV serotypes 1, 2, 3, 5, 7F, 19 according to the standardised multiplexed opsonophagocytic assay (OPA) as described [21] with recent modifications as outlined in their on-line protocol (http://www.vaccine.uab.edu/UAB-MOPA.pdf). The opsonic titre for each serotype will be determined by the dilution of serum that kills 50% of the bacteria. The laboratory where these assays will be performed is yet to be finalised.

6.2.3Memory B-cell assays: Serotype-specific memory B-cell responses post-primary and pre- & post- booster immunisation will be assessed by culturing PBMCs with polyclonal stimuli CpG and Pokeweed Mitogen for 5 days to activate memory B cells as described.[22] After culture these cells will be seeded onto 96 well filter plates that have been pre-coated with polysaccharide antigens for serotypes 6B, 7F, 14 and 19F. Serotype-specific memory B cells will be detected by the formation of spots by ELISpot reader, which will indicate excreted antibody to the antigen, with each spot representing one cell.

6.2.4 T-cell immunity: To assess T-cell responses, PBMC ($1x10^6$ cells/mL) will be resuspended in RPMI/5% HI-AB serum and stimulated with CRM_{197} (10µg/ml), PspA (5µg/ml), CbpA (10µg/ml), Ply (5µg/ml), and PHA (1µg/ml) for 96 hrs of culture (48 hrs for PHA). Cytokines will be measured in supernatants by Luminex, including IL-5, IL-13, IL-10, IFNγ, IL-6, TNFα, IL-9, IL-

17, IL-12, IL-1β and IL-23. We have determined that these cytokines are readily detectable at the protein level in the majority of infants ≥3 months of age.

6.2.5 Bacteriology: Standard procedures established at PNGIMR for bacterial isolation from per nasal swabs will be used.[23,24] NS will be stored at -70°C within 24 hours. 10 µl of STGGB will be cultured on selective media for pneumococci and *H. influenzae* and semi-quantitative colony counts of each species of interest recorded.[16] Two colonies each of pneumococcus and mucoid (encapsulated) strains of Hi will be selected for serotyping using the Quellung reaction and an additional colony if morphologically distinct. One NTHi colony will be selected for confirmatory tests and beta-lactamase production. Antimicrobial susceptibility will be done on 1 pneumococcal isolate (and another isolate if morphologically different or a different serotype) and mucoid strains of Hi. Polymerase chain reaction (PCR) methods for serotyping may be used where appropriately validated including for serotype 6C in any colonies typed as 6A by the Quellung reaction[25]. Samples will be stored for later characterisation of viral respiratory pathogens via multiplex PCR.

6.3 ALRI hospitalisation data collection: To date we have collected data up to age 18 months on children who participated in the neonatal PCV study. Parents keep their children's health books carefully as these are needed to obtain health care. We will check all health books for details of hospitalisations and if hospitalised we will examine medical records. We will also check hospitalisations at Goroka Hospital routinely documented on the paediatric ward admissions book and more recently on a computer database.

7. Statistics

7.1 Sample size: The number of subjects planned to be enrolled. In multicentre trials, the numbers of enrolled subjects projected for each trial site should be specified. Reason for choice of sample size, including reflections on (or calculations of) the power of the trial and clinical justification.

The sample size for the children who have previously received PPV at approximately 9 months of age will be limited by the number of children enrolled in the original neonatal pneumococcal conjugate vaccine who will still be available for follow-up and this is estimated to be approximately 75 children in each of the 3 groups (i.e. 7V-PCV at 0-1-2 months and PPV at 9 months of age; 7V-PCV at 1-2-3months and PPV at 9 months of age; or PPV alone at 9 months of age). We intend to enrol 150 children of the same age who have never received PPV vaccine (controls). Power calculations are based on antibody distributions at 18 months of age in the PCV study as there are no data on serotype-specific antibody levels at 3-5 years of age in PNG children with current assays. Given a standard deviation in log GMC concentrations of 1.25, 75 children in each treatment group and 150 controls would provide 80% power at a 0.05 significance level to detect a 64% difference in serotype GMC concentrations between groups. This sample size would also provide 80% power to detect 16 to 20% differences in the proportion of children with IgG ≥ 1 ug/mL depending on the antibody prevalence for each serotype e.g. if 10% ≥1ug/mL in controls then able to detect 16% difference in each PPV group (26%), and if 50% sero-protected then able to detect a 20% difference in sero-protection rates. For non-7V PCV serotypes, if there are no differences between the PPV groups, then these data can be combined to give a sample size of 225 in the treatment (PPV) group and would provide 80% power to detect 44% differences in the GMC and 11% to 15% differences in the seroprotection rates.

7.2 Data analysis: A description of the statistical methods to be employed, including timing of any planned interim analysis(ses).

All serum antibody concentrations and opsonophagocytic titres will be log transformed for statistical analysis and geometric mean concentrations (GMC) and geometric mean titres (GMT)

calculated with 95% confidence intervals. As the age range (3-5 years) at time of follow-up for previous PCV study participants will be broad (because they were enrolled over a 3-year period), we will control for age in our analyses. Further analyses examining the association of different factors such as priming PCV group (for relevant serotypes), urban/rural etc with response will use multiple regression methods: linear when IgG response, OPA, memory B-cell numbers or cytokine level is the response, logistic when carriage or cytokine detection is the response, Cox when time to first hospital admission is the response, or Poisson when pneumonia admission rate or is the response.

Outcomes and significance

Invasive and mucosal pneumococcal diseases in early childhood are a major global health burden. The potential advantages of use of PPV in addition to PCV immunisation in infancy include enhancement of coverage of important IPD serotypes not included in 10- or 13-valent PCV such as serotype 2, boosting of PCV serotypes to enhance long-term immunity and relative lower vaccine costs. Concerns regarding immunological hypo-responsiveness following polysaccharide vaccines (without evidence of clinical significance) means that it is critical to demonstrate duration of protective antibodies and lack of interference with induction of B-cell memory following pneumococcal polysaccharide vaccine. Detailed cellular immune studies are essential to further our understanding of mechanisms of actions of bacterial conjugate vaccines. The expertise gained from this study will help enhance collaboration with groups in other countries in the region. This proposed study will have important implications for the development of pneumococcal immunisation policy in PNG and other non-industrialised countries and directly inform policy in this area. The results of this study will be particularly relevant for the implementation of the GAVI program for the use of PCV in infants in PNG and other developing countries. Ultimately it is hoped that this strategy will impact on the 2 million deaths occurring each year from pneumonia in young children.

8. Ethics

The study will be conducted according to ICH guidelines of Good clinical practice and will be approved by the institutional review boards of the PNG Institute of medical research, Princess Margaret Hospital for Children as well as the Medical research Advisory Committee of Papua New Guinea. Community consultation regarding the study has already commenced and discussion with previous neonatal PCV study participants about the possibility of being involved in the study has been positive.

References

1. The world's forgotten children. Lancet 2003;361:1.
2. Shann F, et al. Vaccines for children in rich and poor countries. Lancet 1999;354 Suppl 2:SII7-11.
3. Kakazo M, et al. Mortality rates and the utilization of health services during terminal illness in the Asaro Valley, Eastern Highlands Province, Papua New Guinea. P N G Med J 1999;42:13-26.
4. Barker J, et al. Pneumonia in children in the Eastern Highlands of Papua New Guinea: a bacteriologic study of patients selected by standard clinical criteria. J Infect Dis 1989;159:348-52.
5. Lehmann D, et al. Aetiology and clinical signs of bacterial meningitis in children admitted to Goroka Base Hospital, Papua New Guinea, 1989-1992. Ann Trop Paediatr 1999;19:21-32.
6. Richmond P, et al. Effect of neonatal and early immunisation with heptavalent pneumococcal conjugate vaccine on morbidity and pneumonia in Papua New Guinea. In:

Sixth International Symposium of Pneumococci and Pneumococcal Diseases; 2008; Reykjavik, Iceland; 2008. p. P345-6.
7. Riley ID, et al. Pneumococcal vaccine prevents death from acute lower-respiratory-tract infections in Papua New Guinean children. Lancet 1986;2:877-81.
8. Cutts FT, et al. Efficacy of nine-valent pneumococcal conjugate vaccine against pneumonia and invasive pneumococcal disease in The Gambia: randomised, double-blind, placebo-controlled trial. Lancet 2005;365:1139-46.
9. Wright PF, et al. Clinical studies of pneumococcal vaccines in infants. II. Efficacy and effect on nasopharyngeal carriage. Rev Infect Dis 1981;3 Suppl:S108-12.
10. Pomat WS, et al. Immunoglobulin G antibody responses to polyvalent pneumococcal vaccine in children in the highlands of Papua New Guinea. Infect Immun 1994;62:1848-53.
11. O'Brien KL, et al. Combined schedules of pneumococcal conjugate and polysaccharide vaccines: is hyporesponsiveness an issue? Lancet Infect Dis 2007;7:597-606.
12. Malley R, et al. CD4+ T cells mediate antibody-independent acquired immunity to pneumococcal colonization. Proc Natl Acad Sci U S A 2005;102:4848-53.
13. Lipsitch M, et al. Are anticapsular antibodies the primary mechanism of protection against invasive pneumococcal disease? PLoS Med 2005;2:e15.
14. Lehmann D, et al. Safety and immunogenicity of two Haemophilus influenzae type b polysaccharide-tetanus toxoid conjugate vaccines (PRP-T) given with diphtheria-tetanus-pertussis vaccine to young Papua New Guinean children. P N G Med J 2001;44:6-16.
15. van den Biggelaar AH, et al. Neonatal pneumococcal conjugate vaccine immunization primes T cells for preferential Th2 cytokine expression: A randomized controlled trial in Papua New Guinea. Vaccine 2009;27:1340-7.
16. Lehmann D, et al. Bacterial and viral etiology of severe infection in children less than three months old in the highlands of Papua New Guinea. Pediatr Infect Dis J 1999;18:S42-9.
17. Lehmann D, et al. Susceptibility of pneumococcal carriage isolates to penicillin provides a conservative estimate of susceptibility of invasive pneumococci. Pediatr Infect Dis J 1997;16:297-305.
18. Gratten M, et al. Colonisation of Haemophilus influenzae and Streptococcus pneumoniae in the upper respiratory tract of neonates in Papua New Guinea: primary acquisition, duration of carriage, and relationship to carriage in mothers. Biol Neonate 1986;50:114-20.
19. Leach A, et al. Bacterial colonization of the nasopharynx predicts very early onset and persistence of otitis media in Australian Aboriginal infants. Pediatr Infect Dis J 1994;13:983-9.
20. Concepcion NF, et al. Pneumococcal type 22f polysaccharide absorption improves the specificity of a pneumococcal-polysaccharide enzyme-linked immunosorbent assay. Clin Diagn Lab Immunol 2001;8:266-72.
21. Burton RL, et al. Development and validation of a fourfold multiplexed opsonization assay (MOPA4) for pneumococcal antibodies. Clin Vaccine Immunol 2006;13:1004-9.
22. Clutterbuck EA, et al. Serotype-specific and age-dependent generation of pneumococcal polysaccharide-specific memory B-cell and antibody responses to immunization with a pneumococcal conjugate vaccine. Clin Vaccine Immunol 2008;15:182-93.
23. Gratten M, et al. The bacteriology of acute pneumonia and meningitis in children in Papua New Guinea: assumptions, facts and technical strategies. P N G Med J 1991;34:185-98.
24. Montgomery JM, Lehmann D, Smith T, Michael A, Joseph B, Lupiwa T, et al. Bacterial colonization of the upper respiratory tract and its association with acute lower respiratory tract infections in Highland children of Papua New Guinea. Rev Infect Dis. 1990; 12(Supplement 8):S1006-16.
25. Hare KM, et al. "Dodgy 6As": differentiating pneumococcal serotype 6C from 6A by use of the Quellung reaction. J Clin Microbiol 2009;47:1981-2.
